# A Permeability Study of O_2_ and the Trace Amine *p*-Tyramine through Model Phosphatidylcholine Bilayers

**DOI:** 10.1371/journal.pone.0122468

**Published:** 2015-06-18

**Authors:** Bryan W. Holland, Mark D. Berry, C. G. Gray, Bruno Tomberli

**Affiliations:** 1 Department of Biological Sciences, University of Calgary, Calgary, Alberta, Canada; 2 Department of Chemistry, Brandon University, Brandon, Manitoba, Canada; 3 Department of Physics, University of Guelph, Guelph, Ontario, Canada; 4 Department of Physics, Capilano University, North Vancouver, British Columbia, Canada; London, UNITED KINGDOM

## Abstract

We study here the permeability of the hydrophobic O_2_ molecule through a model DPPC bilayer at 323K and 350K, and of the trace amine *p*-tyramine through PC bilayers at 310K. The tyramine results are compared to previous experimental work at 298K. Nonequilibrium work methods were used in conjunction to simultaneously obtain both the potential of mean force (PMF) and the position dependent transmembrane diffusion coefficient, *D*(*z*), from the simulations. These in turn were used to calculate the permeability coefficient, *P*, through the *inhomogeneous solubility-diffusion* model. The results for O_2_ are consistent with previous simulations, and agree with experimentally measured *P* values for PC bilayers. A temperature dependence in the permeability of O_2_ through DPPC was obtained, with *P* decreasing at higher temperatures. Two relevant species of *p*-tyramine were simulated, from which the PMF and *D*(*z*) were calculated. The charged species had a large energetic barrier to crossing the bilayer of ~ 21 kcal/mol, while the uncharged, deprotonated species had a much lower barrier of ~ 7 kcal/mol. The effective *in silico* permeability for *p*-tyramine was calculated by applying three approximations, all of which gave nearly identical results (presented here as a function of the pK_*a*_). As the permeability value calculated from simulation was highly dependent on the pK_*a*_ of the amine group, a further pK_*a*_ study was performed that also varied the fraction of the uncharged and zwitterionic *p*-tyramine species. Using the experimental *P* value together with the simulated results, we were able to label the phenolic group as responsible for the pK_*a*1_ and the amine for the pK_*a*2_, that together represent all of the experimentally measured pK_*a*_ values for *p*-tyramine. This agrees with older experimental results, in contrast to more recent work that has suggested there is a strong ambiguity in the pK_*a*_ values.

## Introduction

The primary biological function of a cytoplasmic membrane is to act as a selectively permeable barrier. For larger molecules, the energy required to cross the lipid bilayer is high and therefore passive diffusion is often too slow to meet cellular needs. For these molecules specific proteins have evolved to control their concentrations, and are extensively studied in the literature [1, 2]. There are many molecules, however, that permeate the cytoplasmic membrane primarily via simple diffusion, e.g. O_2_ and CO_2_, and it is this set of molecules with which the current work is primarily concerned. For most purposes, molecules can be categorized using the Lipinski ‘rule of five’ or related rules [3] as either permeable (small and hydrophobic) or impermeable (large or hydrophilic), but for some molecules of intermediate size or hydrophilicity, passive diffusion is still a biologically relevant form of transport across the membrane (it has been stated that most organic molecules with molecular weights up to 1000 g/mol permeate through the bilayer directly [4]). The trace amine *p*-tyramine (‘tyramine’ herein), derived from tyrosine and involved in the regulation and release of the catecholamines epinephrine, norepinephrine and dopamine [5], may also fall into this category of unassisted transport.

**Fig 1:**
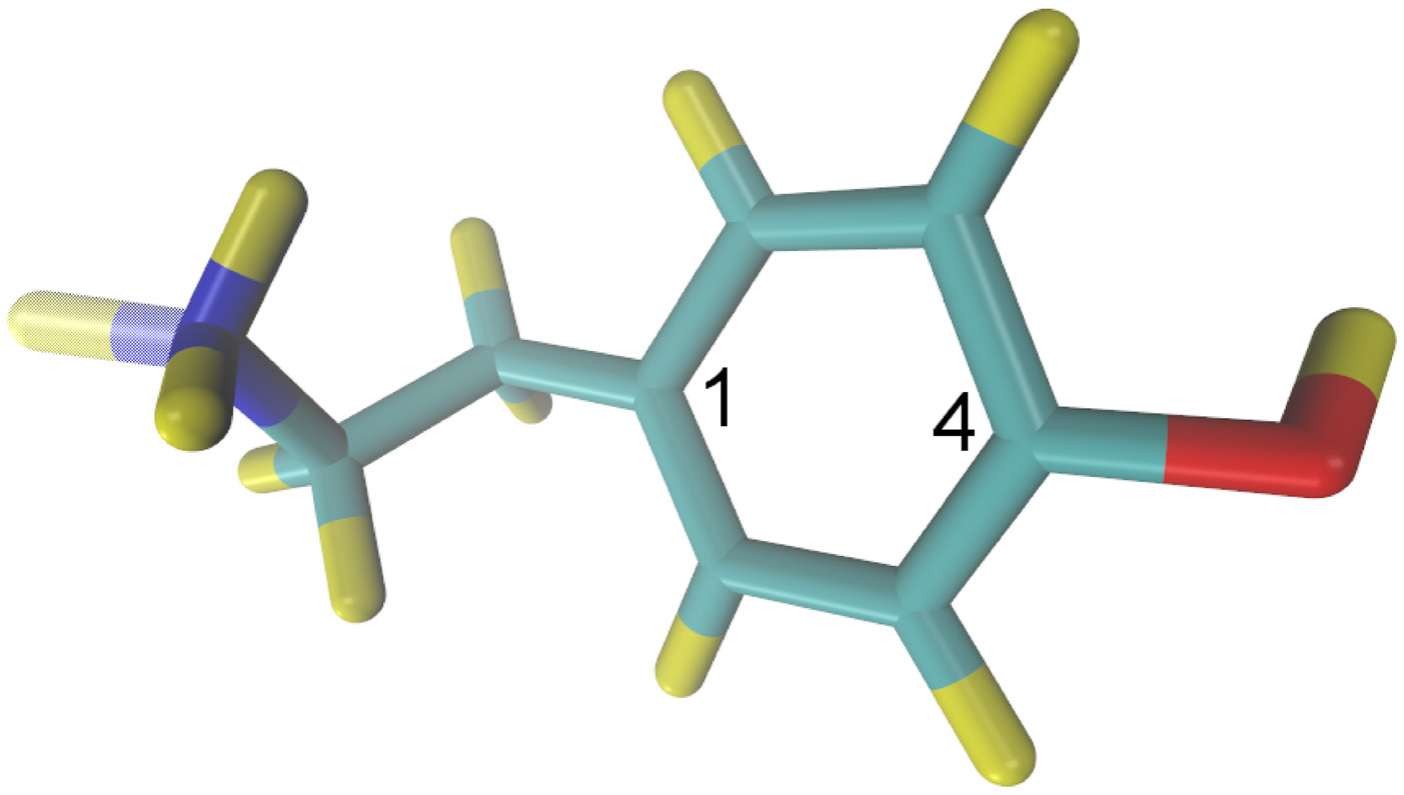
‘Licorice’ model of tyramine. Yellow = H, light blue = C, blue = N and red = O. One hydrogen on the amine is partially transparent to show the two moeities simultaneously: positively charged ${{\text{NH}}_{3}}^{+}$ and neutral NH_2_. Numbers ‘1’ and ‘4’ label the C1 and C4 carbons of the phenol respectively.

Trace amines are neuronally synthesized [5] and have known neuro-regulatory roles in the nervous systems of both vertebrates and invertebrates. In humans, trace amines have been implicated in various neurologic and psychiatric disorders [6–8]. Tyramine (neutral form C_8_H_11_NO, molecular weight = 137.2 g/mol) is an intermediate sized molecule having non-zero fractions of the uncharged, protonated and zwitterionic forms at physiologic pH (Fig 1 shows molecular structures for the uncharged and protonated forms of tyramine). The identification of a sub-set of G-protein coupled receptors (GPCR) that are selectively activated by trace amines such as tyramine [9, 10] has sparked a resurgence of interest in this class of chemicals. Unlike most GPCRs, evidence suggests that the Trace Amine-Associated Receptors (TAAR), at which tyramine is a ligand, are poorly translocated to the cell membrane. Rather, these TAARs appear to remain associated with intracellular membranes [11]. Older evidence suggests that extracellular levels of tyramine exist in a steady state, determined by the relative rates of synthesis and degradation [12–14]. This suggests that membrane diffusion may be a dominant factor in determining extracellular tyramine [5, 15], unlike more traditional monoamine neurotransmitters whose extracellular levels are determined by the rate of exocytotic release [16]. Such non-exocytotic release is further supported by recent studies reporting an apparent decrease in tyramine release from nerve terminal preparations under conditions in which exocytosis is stimulated [17]. Further, these studies reported increased diffusion of tyramine across lipid bilayers in the absence of membrane proteins, in contrast with dopamine and noradrenaline [17]. Finally, no transporter has yet been identified for tyramine and hence passive diffusion has been proposed to be the primary mode of transport though the cytoplasmic membrane [5, 17].

As part of our studies to further characterize and understand the mechanisms involved in controlling extracellular trace amine levels, we use previously developed computer simulation techniques that predict the energetics and dynamics of molecular passage across lipid bilayers, and report here the validation of such methods against experimentally determined permeability coefficients. As a comparator we have also determined the permeability of O_2_, a molecule known to primarily cross the membrane by diffusion due to its neutrality and small size, and that is clearly important in the physiology of numerous organisms. These properties make oxygen an excellent test of our methods and theories. Furthermore, while there have been numerous studies on the permeability of oxygen through lipid bilayers [18–20], none have studied the temperature dependence of the oxygen permeability. Therefore, we have simulated O_2_ permeation through DPPC bilayers at 323 K and 350 K and compare our results to those from previous simulations.

The permeability coefficient, *P*, across a complex, yet symmetric medium such as a bilayer can be calculated using the *inhomogeneous solubility-diffusion* (ISD) model, originally proposed by Marrink and Berendsen [21] and that has the form:

$$\begin{array}{c} P={\left[\int_{0}^{L}dz\frac{e^{\beta w(z)}}{D(z)}\right]}^{-1}\;, \end{array}$$ (1)

where *β* = (*k*_*B*_ *T*)^−1^ and *k*_*B*_ is Boltzmann’s constant, *w*(*z*) is the *potential of mean force* (PMF) along the reaction coordinate *z* (the direction perpendicular to the medium’s surface), *D*(*z*) ≡ *D*_*zz*_(*z*) is the local diffusion coefficient for the *z* direction (along the reaction coordinate), i.e. the *zz* diagonal component of the diffusion tensor, and *L* is the width of the medium. For brevity, we will herein refer to *D*_*zz*_(*z*) as simply *D*(*z*). Obtaining PMF profiles from simulations has been a subject of intense interest for many years [22], and recent advances in statistical mechanics [23–26] have also allowed the use of nonequilibrium trajectories to obtain them. In this work we employ the fast converging bidirectional method developed by Kosztin *et al*. [25] to obtain both the reversible work, or change in free-energy, Δ*F*, and the mean dissipative work, ⟨*W*_*d*_⟩, using trajectories steered at finite velocity from an initial equilibrium state A to a final equilibrium state B, and again in reverse. The desired quantities are related to the average of the forward and reverse work distributions through the following simple formulae:

$$\begin{array}{c} \Delta F=\frac{\left\langle W_{F}\right\rangle -\left\langle W_{R}\right\rangle }{2}\;, \end{array}$$ (2)

$$\begin{array}{c} \left\langle W_{d}\right\rangle =\frac{\left\langle W_{F}\right\rangle +\left\langle W_{R}\right\rangle }{2}\;. \end{array}$$ (3)

Work by our group has confirmed that this *forward-reverse* (FR) method accurately determines the PMF [27]. Furthermore, we have shown that the PMF profiles may be constructed from small oscillations in the forward and reverse directions with a steady drift across the system, resulting in completed PMFs along the reaction coordinate of interest while only travelling overall in the forward direction from A → B; we call this the *oscillating forward-reverse* method (OFR). An interesting corollary of our studies shows that to obtain a ⟨*W*_*d*_⟩ that—to a good approximation—is linearly proportional to the speed of the particle, *v*, the steering protocol must be carefully implemented to control the particle’s path [28]. To this end, a constraint (perfectly rigid steering) must be used rather than a restraint (steering with a spring of finite stiffness). We call such steering the *dynamic constraint protocol* (DCP) to differentiate it from the more typically used static (or equilibrium) protocols (SCP), and from protocols involving restraints (SRP and DRP). With ⟨*W*_*d*_⟩ thus determined, *D*(*z*) can be calculated using the Stokes-Nernst-Einstein relation,

$$\begin{array}{c} \frac{d\left\langle W_{d}\right\rangle \left(z\right)}{dz}=\frac{k_{B}Tv}{D(z)}\;, \end{array}$$ (4)

where *v* is the particle speed at *z*. We also use the Brownian dynamics fluctuation-dissipation theorem (BD-FDT) [26] in lieu of Eq 2 to calculate the PMF from the OFR results; it is an expression for the PMF that correctly biases work values used in the averaging with a Boltzmann-like weighting. Essentially, nonequilibrium paths with higher work values are exponentially less probable to occur in an equilibrium system, and are thus given less weight in the average. The BD-FDT has the form:

$$\begin{array}{c} \Delta F=\frac{\left\langle e^{-\beta W_{F}/2}\right\rangle }{\left\langle e^{-\beta W_{R}/2}\right\rangle }\;. \end{array}$$ (5)

The theory we have outlined here and that implements Eqs 1 and 3–5, allows the permeability of a species to be determined efficiently from a small number of simulated molecular dynamics trajectories. Applying these methods to O_2_ and tyramine are the focus of the results presented in this work. There are also equilibrium methods that are capable of calculating the PMF and *D*(*z*) from the same simulation trajectories [29, 30], and there have been various other equilibrium algorithms used in part to calculate membrane permeability rather than the nonequilibrium method we use, e.g., unbiased dynamics [21], Widom particle insertion [21], force constraints [18, 21, 31], umbrella sampling [32], the adaptive biasing force method [33] and bias exchange metadynamics [34]. No direct comparison of the methods is performed here, although this will be the focus of future work.

## Materials and Methods

### Experimental

Experimental determination of the permeability coefficient for tyramine passage across a lipid bilayer was previously determined using the commercially available Fluorosome system (GL Synthesis Inc., Worcester MA), as described in [17].

Briefly, fluorescence was monitored using a Spectramax M2 plate-reader (Molecular Devices, Sunnyvale, CA) operating in kinetic mode at an excitation wavelength of 494 nm and emission wavelength of 523 nm. Baseline fluorescence of Fluorosomes in the manufacturer’s supplied H-buffer (pH adjusted to 7.4) was determined at 1 s intervals for 50 s, followed by the addition of 93.8 mM tyramine hydrochloride (Sigma Aldrich, Oakville, ON). Post-addition fluorescence was followed at 2 s intervals for a further 300 s. All assays were conducted at a temperature of 298 ± 1 K.

Post-addition data was fit to a one-phase exponential decay function using GraphPad Prism 5.0 software (GraphPad, La Jolla, CA), and curve parameters imported into the Fluorosome manufacturers supplied software algorithm to obtain a permeability coefficient from seven independent determinations.

### Computer simulations

All-atom simulations were performed using the simulation package NAMD 2.7 [35], with an augmented version of the TclForces package in order to apply the DCP, as previously discussed in [28]. The CHARMM27 force-field [36] was used for all simulations, although when a bilayer was present the CHARMM36 lipid force-field [37] was used to represent the phospholipids. Unless otherwise noted, water was modeled using the modified TIP3P model [38] standard in NAMD. This water model was chosen in order to enable a consistent comparison against previously published results [18–20] that also used three-point water models, and because the force-field used was parameterized using TIP3P—meaning that this combination is more likely to get accurate results for the PMF. This model was used even though the *D* of bulk water using TIP3P is about twice that found experimentally [39], as within the framework of the ISD model, the diffusion coefficient is important, but the energetics are exponentially more so.

Previously, the permeability of H_2_O across a dipalmitoylphosphatidylcholine (DPPC) bilayer was determined using the OFR method [28], but as a separate test it was also of interest to determine the permeability of a small nonpolar molecule and compare it to previous results in the literature. To this end, a single O_2_ molecule was steered across a DPPC bilayer using the OFR method under NPT conditions. Temperature control was performed through the use of Langevin dynamics with a damping coefficient of 5 ps^−1^, while the anisotropic barostat used was the Langevin piston standard in NAMD. As no parameters exist for O_2_ in the CHARMM27 force field, the values for the Lennard-Jones potential were chosen to correspond with those from Shinoda *et al*. [19]: *ϵ*_*OO*_ = 0.12 kcal/mol and *σ*_*OO*_ = 3.4 Å. The value of the harmonic force constant for the double bond was taken from experimentally derived tables [40] to be *k*_*O*_2__ = 1694 kcal/(mol ⋅ A^2^) and the partial charges were set to zero.

Also, to compare with previous work, the simulations were carried out at 323 K and 350 K with a pressure of 1 atm. The previous authors likely chose these temperatures to stay above the gel to liquid-crystal phase transition for DPPC, experimentally determined to be 314.5 K [41]. Recent work by Schubert *et al*. [42] reported a first-order phase transition occuring at 321 K for DPPC in the CHARMM36 force-field, so both of the current systems should also be in the biologically relevant liquid-crystalline phase. Bond lengths between hydrogen and heavy atoms were constrained using NAMD’s *rigidBonds* parameter, allowing an integration time step of 2 fs. The system consisted of 32 DPPC phospholipid molecules per leaflet, and a total of 1945 water molecules to fully hydrate the bilayer with ∼ 30.4 waters per DPPC. The process was repeated six times at both temperatures from uncorrelated, equilibrated states in order to perform *multiple path sampling* (see [28] for details); the output file for one run at 350 K was corrupted for unknown reasons and thus not included in the averaging. The O_2_ molecule was steered along the reaction coordinate using the DCP, such that the distance between the center of mass (COM) of the O_2_ and that of the bilayer, *z*_*COM*_, proceeded exactly along a path described by:

$$\begin{array}{c} z_{COM}\left(t\right)=z_{0}+v_{d}t+A\sin\left(\omega t\right);\;\;\omega =\frac{\pi nv_{d}}{A}. \end{array}$$ (6)

Here *v*_*d*_ is the drift velocity, *A* is the oscillation amplitude, *ω* is the oscillation frequency, and *n* is the approximate number of samples taken. The molecule was allowed to move freely in the plane parallel to the surface of the bilayer (x-y plane); this is a standard sampling technique with bilayers that allows the molecule to interact with many more lipids than it would if constrained to move along the vector between the two COMs. It was shown previously [28] that to obtain an accurate *D*(*z*) there is a limit for the average oscillation speed of the particle, *v*_*av*_ = 2*nv*_*d*_, beyond which the work dissipated into the surrounding medium is no longer linear with respect to the speed. This limiting value is refered to here as the *critical speed*, *v*_*c*_. Previous tests showed that a *v*_*c*_ ≈ 800 Å/ns is a conservative estimate for this system [28], and so a *v*_*av*_ = *v*_*c*_/4 = 200 Å/ns was used to stay well below the limiting value. This was accomplished by using *v*_*d*_ = 2 Å/ns, *A* = 1 Å and *n* = 100, leading to a total simulated time for each run of ∼ 20 ns.

Unlike O_2_, multiple states of tyramine can co-exist. As both the amine and phenolic groups have a pK_*a*_ ≳ 9.3 [43–45], the majority of tyramine exists in its fully protonated form within the human body (pH ∼ 7.4). When the very low bilayer permeability of ionic species is considered, however, there could still be enough deprotonated tyramine to dramatically affect the permeability coefficient across the bilayer. Therefore to properly calculate the permeability of tyramine, both positively charged (${{\text{NH}}_{3}}^{+}$ and OH moieties, *tyr* ^+^) and uncharged (NH_2_ and OH moieties, *tyr*) species were simulated. Two other charge states exist for tyramine, being zwitterionic (with ${{\text{NH}}_{3}}^{+}$ and O^−^ moieties, *tyr* ^+/−^) and negatively charged (with NH_2_ and O^−^ moieties, *tyr* ^−^). Given the reported pK_*a*_ values, the zwitterionic species is in a dipolar state with a maximum fractional population of 8 × 10^−3^ at a pH of 7.4, and its omission from this work is justified as follows. Khavrutskii *et al*. [46] simulated the permeability of Na^+^, Cl^−^, and their ion pair, and found the pair to have a higher energetic barrier than either ion on its own. The *generalized Born* (GB) model [47, 48] describes an approximation for the difference in free-energy of *n* charges that have been transferred from medium *w* to medium *b*,

$$\begin{array}{c} \Delta G=\frac{1}{2}(\frac{1}{\epsilon_{b}}-\frac{1}{\epsilon_{w}})(\sum_{i=1}^{n}\frac{q_{i}^{2}}{a_{i}}+\sum_{i=1}^{n}\sum_{j\neq i}^{n}\frac{q_{i}q_{j}}{r_{ij}})\;. \end{array}$$ (7)

Here, *q*_*i*_ and *q*_*j*_ represent a charge pair, *ϵ*_*w*_ is the dielectric constant for water, *ϵ*_*b*_ is the dielectric constant at the center of the bilayer, *a*_*i*_ is the *Born radius* of each charge, and *r*_*ij*_ is the distance between each charge pair. From Eq 7, the following ratio can be derived for the free-energy of transferring a pair of opposite and equal charges, relative to the free-energy of transfering a single positive charge:

$$\begin{array}{c} \frac{\Delta G_{+/-}}{\Delta G_{+}}=\frac{a_{+}+a_{-}}{a_{-}}-\frac{2a_{+}}{r_{+/-}}\;. \end{array}$$ (8)

The ratio of Δ*G*_Na^+^Cl^−^_/Δ*G*_Na^+^_ in [46] is ≃ 1.26. Using Eq 7, with *a* for Na^+^ and Cl^−^ taken from [49] to be 1.80 Å and 1.91 Å, respectively, this gives an average interatomic spacing *r*_+/−_ = 5.28 Å. This agrees well with the second minimum for the interatomic PMF of Na^+^-Cl^−^ in water, found through *ab initio* [50] and classical MD simulations [27, 50]. The Born model states that this ratio should increase with *r*_+/−_, and since *tyr* ^+/−^ has a charge separation closer to 8 Å, a lower bound of Δ*G*_*tyr*^+/−^_ ≥ Δ*G*_*tyr*^+^_ can safely be assumed. Given the low population of *tyr* ^+/−^ combined with the high barrier for *tyr* ^+^ (see ‘Results’), *tyr* ^+/−^ was considered to provide a negligible contribution to the total permeability and was therefore not simulated. Similarly, *tyr* ^−^ was also ignored as it makes up an even more minute fraction of tyramine at a physiological pH.

As tyramine is not a widely simulated molecule, no standard force-field parameters exist for either form. Previous attempts have been made at calculating the permeability of *tyr* ^+^ across a POPC bilayer [51], from which the molecular force-field parameters for this simulation were taken. The uncharged form had no parameters available, however, and so the quantum mechanical software Gaussian 03 [52] was used to calculate the partial charges through the online ‘RESP ESP charge Derive Server’ (RED Server) [53]. The RED Server uses a number of different initial orientations for the molecule, and has been shown to converge well to a reproducible set of partial charges. Additional independent calculations were performed using Gaussian directly with multiple basis sets in order to ensure the reliability of the RED Server results. Regardless, the partial charges supplied for *tyr* by the RED Server were reasonable when considering the individual chemical groups, and are provided in S1 Appendix. Along with the calculated partial charges, standard CHARMM27 values of amino acid-like molecules were used for the Lennard-Jones, bond, angle and dihedral parameters (see the ‘Discussion’ section regarding possible improvements to the parameterization). The systems surrounding the *tyr* and *tyr* ^+^ molecules were nearly identical: a bilayer composed of 207 palmitoyloleoylphosphocholine (POPC), 9548 water molecules and 0.15 M Na^+^-Cl^−^; the *tyr* ^+^ solution contained one extra Cl^−^ to neutralize the system. Both simulations were carried out under NPT conditions with *T* = 310 K and *P* = 1 atm to mimic physiological conditions.

Tyramine is an asymmetric molecule of intermediate size, so it was necessary to determine the rotational relaxation time in order to ensure sufficient sampling of the orientational degrees of freedom. This was accomplished by simulating *tyr* ^+^ in the solution portion of the system—under the same conditions as above—and then calculating the rotational autocorrelation as a function of time, $c_{r}(t)=\langle \hat{\text{r}}(t)\cdot \hat{\text{r}}(0)\rangle$; the molecule was assumed to be stiff enough to use a vector from C_1_ to C_4_ of the phenol group as **r** (see Fig 1)). Also, rotational relaxation of the vectors orthogonal to **r** was assumed to be at least as fast, as **r** points along the longest dimension of the molecule. The characteristic rotational relaxation time, *τ*_*r*_, is then defined as

$$\begin{array}{c} \tau_{r}=\int_{0}^{\infty }c_{r}\left(t\right)dt\;. \end{array}$$ (9)

**Fig 2:**
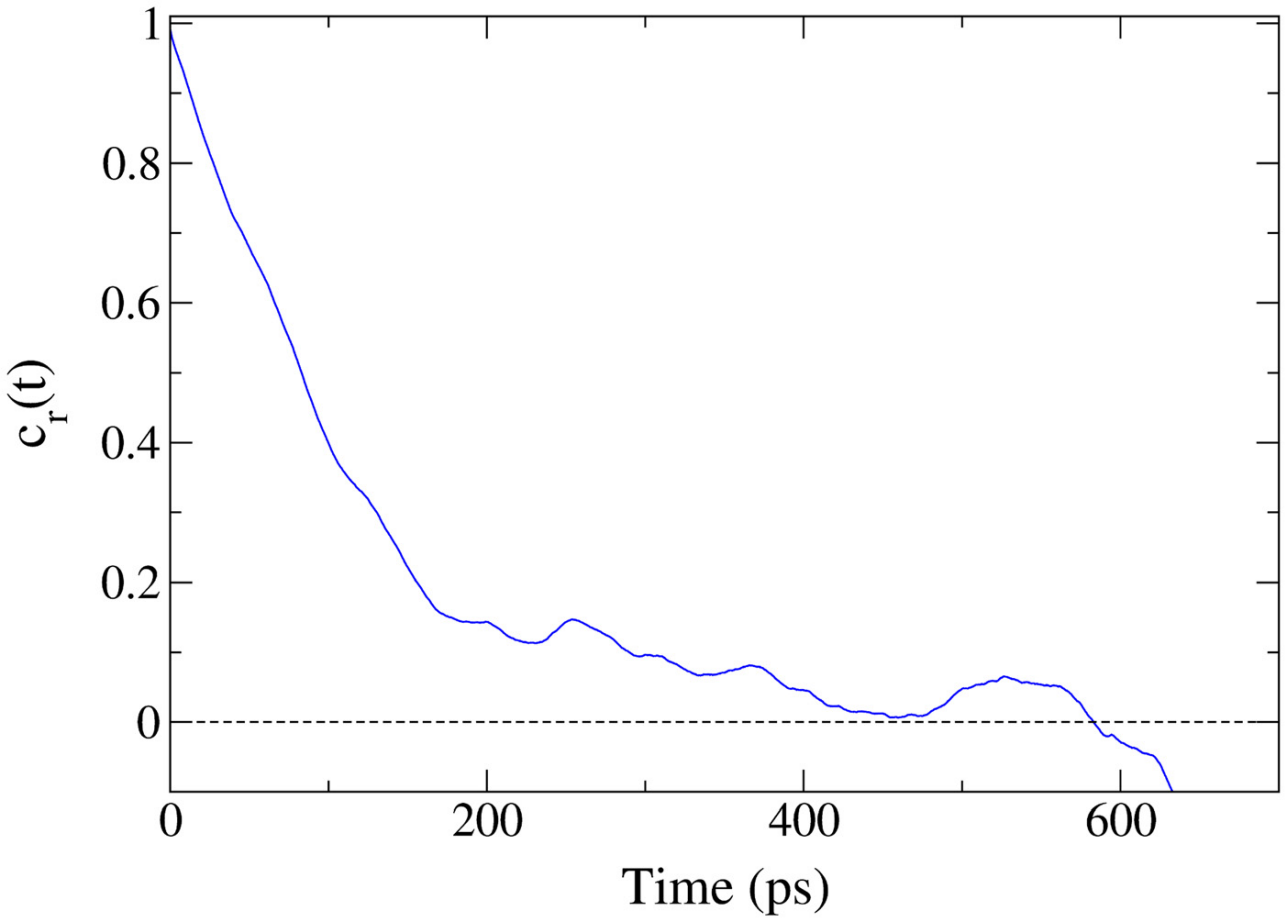
The rotational correlation function, $c_{r}(t)=\langle \hat{\text{r}}(t)\cdot \hat{\text{r}}(0)\rangle$, of *tyr* ^+^ with the COM constrained at ∼ 10 Å outside of a POPC bilayer.

Fig 2 shows *c*_*r*_(*t*) of *tyr* ^+^ for approximately 600 ps. As *c*_*r*_(*t*) theoretically decays to zero at infinity, it is reasonable to use Eq 9 up to the first time that *c*_*r*_(*t*) = 0. Integrating from *t* = 0 → 583 ps, Eq 9 gives *τ*_*r*_ = 117 ps, and it is clear from the plot that most of the rotational correlation is absent after only 200 ps. This information was then used to create the ten starting points for the OFR runs, with the distance between tyramine’s COM and the bilayer’s COM constrained near 40 Å for 10 ns and allowed to diffuse freely in the plane parallel to the bilayer surface, as in the complete runs. Taking the system state at 1 ns intervals as the starting points, the tyramine molecules were separated temporally such that both the rotational orientation and position relative to the plane of the bilayer surface were completely decorrelated. The sampling of tyramine was also informed through *τ*_*r*_ by ensuring there was enough time spent in solution to allow the molecule to rotate. The OFR parameters used in the bilayer permeability simulations were as follows: *v*_*d*_ = 1 Å/ns, *n* = 50, *A* = 2.5 Å. This kept the tyramine in solution for ∼ 5 ns, during which time it had little interaction with the bilayer. For a single run this is likely insufficient, but together with the nine other repeated simulations provides a sufficient amount of rotational sampling. It was assumed that the uncharged species *tyr* has a smaller *τ*_*r*_ in water due to its decreased interaction with the surrounding solution, and could be sampled at least as well as *tyr* ^+^ with the same OFR parameters.

**Fig 3:**
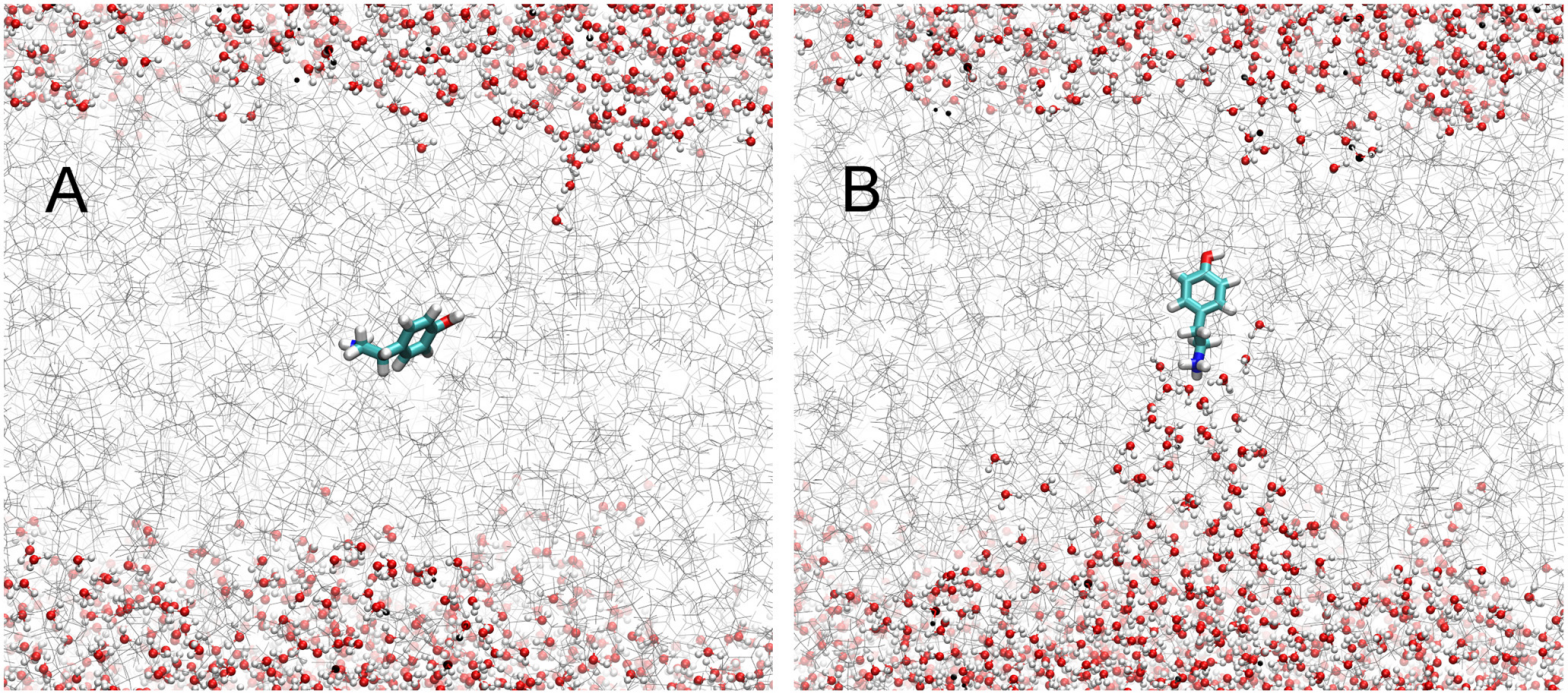
Snapshots of the tyramine/POPC systems, with tyramine near the bilayer center. (A) The unprotonated *tyr* permeates without any water accompaniment, and with no obvious orientational preference. (B) The protonated *tyr* ^+^ never loses the solvation shell around the amine group, and as such always orients along its long axis with the phenolic group leading and the amine trailing. For a more detailed orientation analysis, see [54].

For bilayer interaction, appropriate orientational sampling is justified in a different manner. By allowing the tyramine to orient randomly each time it approached the bilayer in the ten separate simulations, we were able to sample over a wider array of incoming orientations. Although due to its large dipole, *tyr* ^+^ tended to orient itself in a similar manner each time it approached the bilayer, regardless of the original randominized orientation (see Fig 3). The sampling was also helped by using a larger oscillation amplitude of 2.5 Å, forcing the molecule to interact with portions of the bilayer, and then retreat sufficiently to allow for a change in orientation the next time it came into contact with the bilayer. This kept the molecule from becoming stuck in a particular orientation with a metastable energy, a very common problem for sampling in equilibrium based methods. Finally, there exists a highly probable orientation for *tyr* ^+^ within the bilayer [54] and so it is assumed that the most relevant portions of the orientational phase space have been sampled.

All OFR output was analyzed using the OFR Analysis Tool [55] that converts the force data from the nonequilibrium simulations into both averaged work values, and averaged exponentiated work values in both the forward and reverse directions. As in previous work [28], only the results for the exponentiated work values are used along with the BD-FDT (Eq 5) to calculate the PMF, while the averaged work values are used to calculate *D*(*z*) with Eqs 3 and 4. This is because the BD-FDT weighs the importance of the phase space sampled—with regard to the equilibrium free-energy—by applying a Boltzmann weighting to the work values prior to averaging, and does a better job of removing bias added to the simulation through nonequilibrium sampling than the FR method alone. The bin size is a parameter chosen at the time of analysis, and a value of 0.2 Å was used as it is small enough to provide a very smooth PMF curve.

The work values calculated from the individual runs have statistical uncertainties calculated as per [55], which takes into account both autocorrelation (within a bin) and correlation between bins. The final Δ*G* for each bin is then calculated as a weighted average of the individual runs, where the uncertainty acts as the weight; it is important to note that this bin averaging takes place *prior to* the integration that gives the final PMF. As well, all final PMF curves are in turn weighted averages of their respective integrals performed in both directions along the reaction coordinate *z*, where the uncertainty is again used as the weight. At each point, a conservative value for the final uncertainty is then taken to be the average of the uncertainties of the two integrals. This allows for more consistent error bars across the length of the curve, instead of an uncertainty that increases in the direction of integration.

For diffusion, the raw *D*(*z*) values contain a large amount of noise, even after averaging over all individual runs. To reduce the noise, a moving average was applied over 9 data points to smooth the final *D*(*z*) values for each system.

## Results

### Dioxygen permeability across DPPC

**Fig 4:**
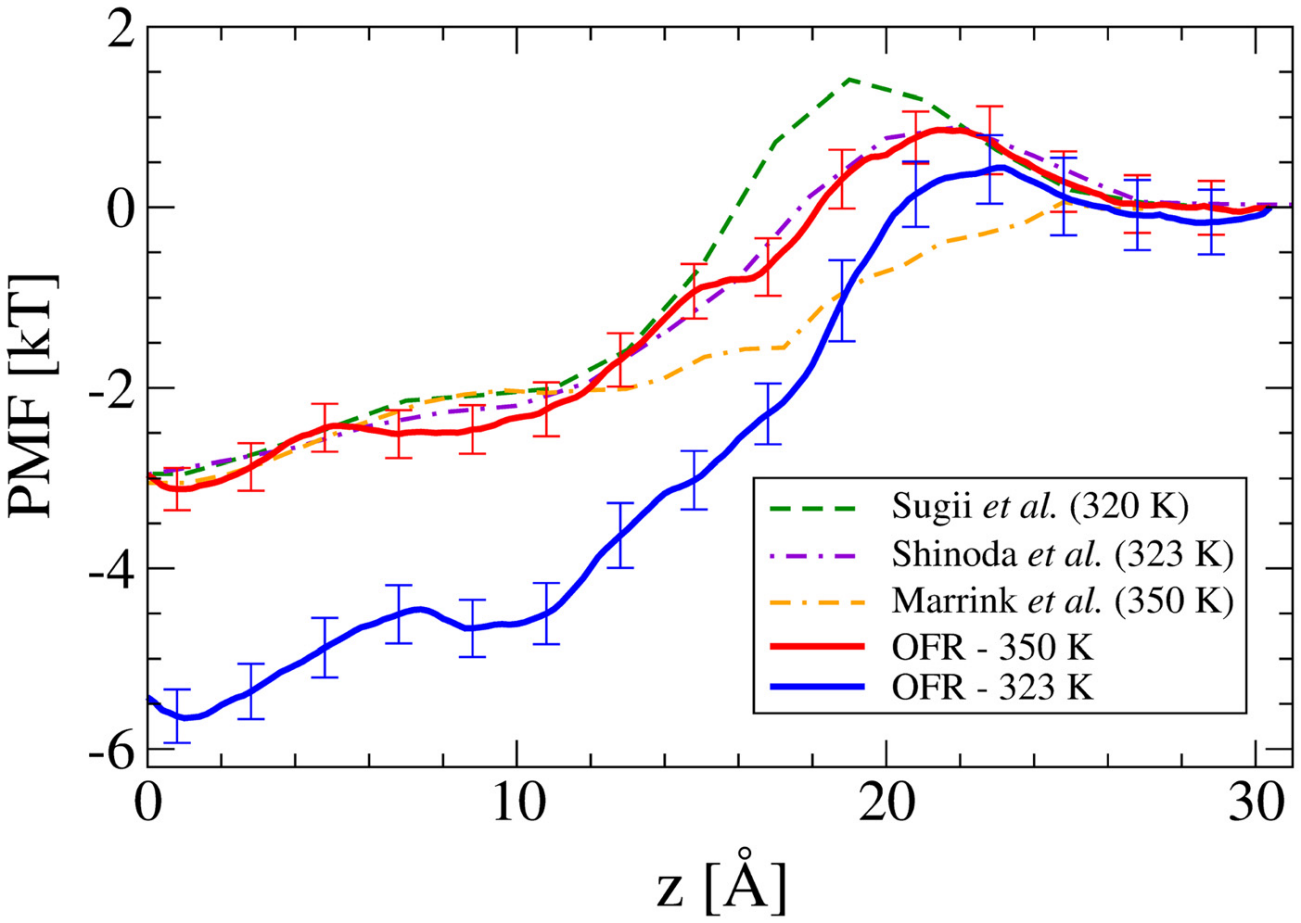
The PMF of O_2_ shown together with previously published curves, in units of kT for the most relevant comparison at different temperatures. The bilayer center is at *z* = 0 and the bulk solution begins at *z* = 30 Å. The error bars represent two standard errors.

Fig 4 shows the two O_2_ curves compared against previous work by Marrink and Berendsen at 350 K [18], Shinoda *et al*. at 323 K [19] and Sugii *et al*. at 320 K [20]. The OFR curve at 350 K agrees with the Marrink and Berendsen curve, other than a barrier to penetration in the headgroup region of the bilayer that is also seen in the other two published works. Our results show a clear variation with temperature, as the size of the hydrophobic well at the bilayer center increases by ∼ 2.5 kT at 323 K, and there is a slight decrease in the headgroup barrier. The difference in well depth becomes most apparent when considering the linear distribution function, *g*(*z*) = exp(−*βw*(*z*)), as O_2_ is nearly twelve times as likely to be found at the bilayer core at 323 K, a dramatic effect considering the temperature difference is only 27 K. Since the solubility of O_2_ in liquid water decreases with increasing temperature [56], this dependence is likely caused by a larger concurrent decrease in its lipophilicity. This results in a *net* reduction in the hydrophobicity of O_2_ for this particular system, and thus reduces the energetic gain of O_2_ reaching the hydrophobic core of the bilayer. The temperature dependence shown in Fig 4 has not been predicted elsewhere, as all three previously published works have near identical well depths when measured in units of *k*_*B*_ *T*. It should be noted that there is ongoing debate in the literature about the temperature dependence of the hydrophobicity of various solutes (e.g. [57, 58]).

**Fig 5:**
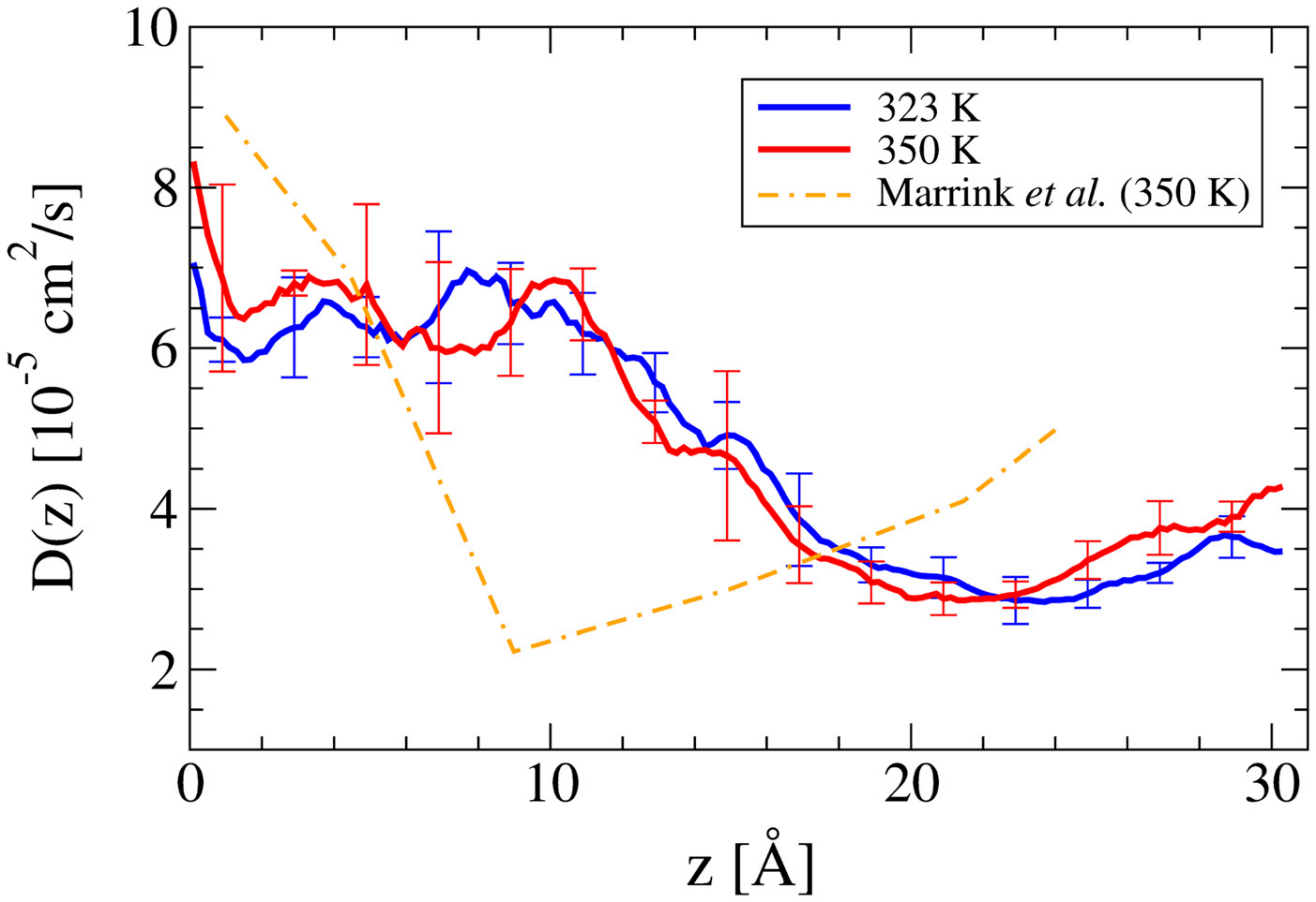
The position dependent diffusion coefficient, D(z), of O_2_ across a DPPC bilayer at 323 K and 350 K with *z* = 0 being the bilayer centre and *z* = 30 Å being in the bulk solution. The error bars represent two standard errors.

Although the PMF shows a strong temperature dependence, the diffusion coefficient (Fig 5) exhibits little difference outside of the measured uncertainties between the two runs. A possible explanation is that although the increase in temperature increases the mobility of the DPPC molecules, the majority of that increase occurs in the plane lateral to the bilayer. As such, there is little change in the local environment as seen by the O_2_ molecule as it moves normal to the bilayer surface. The *D*(*z*) curves for O_2_ are similar to that for an H_2_O molecule permeating a DPPC bilayer [28], which is sensible as diffusion through a hydrophobic environment should be mostly influenced by the molecule’s size and shape, not its polarity.

The permeability values of O_2_ can be calculated by putting the PMF and *D*(*z*) results into Eq 1, and doubling the resistance value calculated to account for the two leaflets of the bilayer. This yields the values *P* = (123 ± 35) cm/s and *P* = (78 ± 19) cm/s, for 323 K and 350 K respectively.

### Tyramine permeability across POPC

#### Experimental

As previously reported, tyramine diffusion across Fluorosome membranes displayed the expected one-phase exponential decay function [17]. The permeability coefficient for this passage at a pH of 7.4 and a temperature of 298 ± 1 K is determined to be (2.26 ± 0.43) × 10^−7^ cm/s, where the uncertainty is taken to be one standard error of the distribution. As the Fluorosome membranes are composed of lipids extracted from egg lecithin, they have a composition of ∼ 45–50% saturated phospholipid, namely DPPC and DSPC. When purified, both of the latter lipids exist as gels at 298 K [41, 59], while the remaining unsaturated lipids (e.g. POPC and DLPC) are in the liquid-crystalline phase [59, 60]. This difference in melting temperatures has been shown to lead to phase separation at room temperature for POPC/DPPC mixtures [61, 62]. The effect of this phase separation for the current system is that tyramine would likely have a much lower permeability through the gel regions comprised of the saturated lipids. Thus, nearly the entire contribution to the measured *P* value occurred through the unsaturated lipids that made up the regions of liquid-disorder. This limits the membrane surface area through which the tyramine may permeate by roughly half, so we argue that the permeability through these regions—regions that are more directly comparable to the simulations results—is roughly twice that of the measured value reported above, *P*_*eff*_ ≈ 4–5 × 10^−7^ cm/s.

#### Computer Simulation

The parameterizations used in this work for both *tyr* and *tyr* ^+^ rely on the accuracy of the density functional method used, along with the similarity of both molecules to the well parameterized amino acid tyrosine. As an accuracy test of these parameterizations, the free-energy of solvation was calculated for both molecules in TIP3P water at 310 K by using the thermodynamic integration methods in NAMD (soft-core potentials and decoupling were used). This resulted in Δ*G*_*tyr*_ = −8.6 kcal/mol and Δ*G*_*tyr*^+^_ = −28.1 kcal/mol. Δ*G*_*tyr*_ is consistent with experimental results [63], the magnitude being ∼ 40% larger than *p*-cresol, a phenol that lacks the amine group of *tyr* and thus has a lower dipole moment. Although the solvation energies are individually reasonable, the ΔΔ*G* gives a pK_*a*_ for the amine of 13.7; this is likely an overestimate considering the lack of parameterization for the molecules in water (see ‘Discussion’). As such, only experimental values for the amine pK_*a*_ are considered throughout the rest of the work.

**Fig 6:**
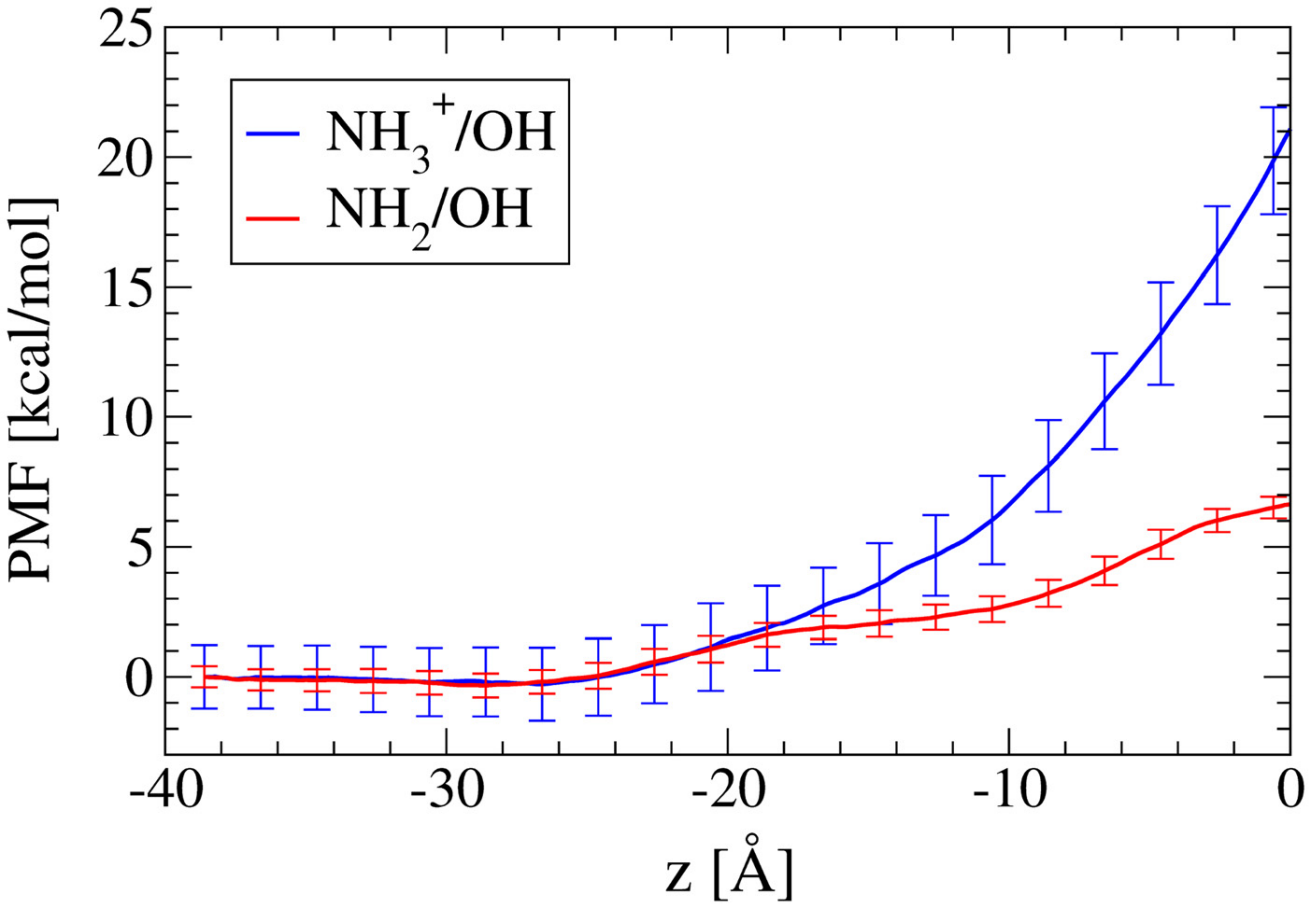
The PMF of *tyr* and *tyr* ^+^ shown together for comparison. Error bars are to two standard errors. Here *z* = −40 Å is the bulk solution andf *z* = 0 is the bilayer centre.

With respect to the bilayer, the diffusive mechanisms for the two tyramine species are quite different. The mechanism for *tyr* is consistent with the diffusive transport of other small molecules of low polarity, e.g. water, where the interaction with solution is weak enough that the molecule can cross the bilayer alone (most of the time). On the other hand, *tyr* ^+^ transport resembles the diffusive mechanism of simple ions crossing a lipid bilayer [64], where water ‘bays’ form that connect the ionic solvation shell to the bulk solution. With simulated electric dipole moments *μ*_*tyr*_ = 2.352 D and *μ*_*tyr*^+^_ = 20.446 D in 0.15 M NaCl solution (measured about their centers of mass in TIP3P water), it is not surprising that the PMF curves for tyramine in Fig 6 are much different from those for O_2_ (*μ* = 0). In fact, *tyr* has a curve very similar to that of a single water molecule (*μ*_TIP3P_ = 2.35 D, [65]) permeating DPPC, but with a slightly lower barrier height at the bilayer center (see [28]). The *tyr* ^+^, however, never loses the hydration shell around the amine group and has a peak barrier height similar to that of monatomic ions at the bilayer center [46, 64, 66].

**Fig 7:**
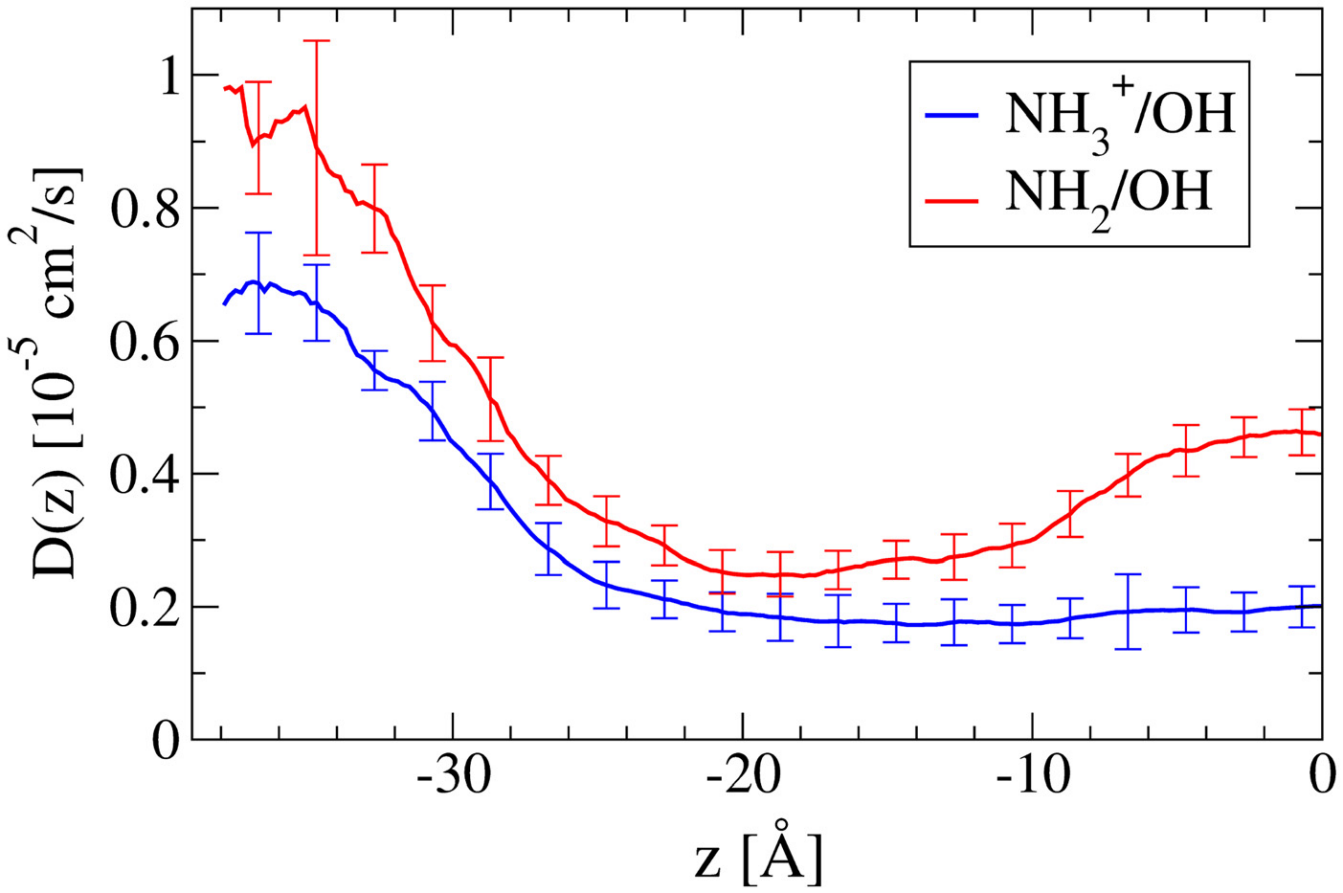
The position dependent diffusion coefficient, D(z), of *tyr* (NH_2_/OH) and *tyr* ^+^ (${{\text{NH}}_{3}}^{+}/\text{OH}$) across a POPC bilayer with the bilayer center at z = 0. The diffusion for *tyr* ^+^ remains relatively constant once inside the bilayer. This is likely due to a water channel being created in the POPC through a strong interaction of water with the ${{\text{NH}}_{3}}^{+}$ group. The water follows *tyr* ^+^ through and reduces its mobility while keeping it in a consistent environment. The *tyr* permeates without water and has mobility characteristics that correlate strongly with the bilayer density, including a densely packed region near the head groups and an area of high free volume at the center of the bilayer where the hydrocarbon tails meet.

The bulk diffusion values, *D*(∞), of *tyr* ^+^ (∼ 0.65 × 10^−5^ cm^2^/s) and *tyr* (∼ 0.9–1 × 10^−5^ cm^2^/s) shown in Fig 7 are about 4–5 times lower than that of O_2_, consistent with the increase in molecular volume. There is a measurable difference between the *D*(∞) values, however, that cannot be explained by their near identical size and shape. The difference in bulk diffusion can be attributed to the *dielectric friction* experienced by ions as they travel through a polar medium [67]; the dielectric friction manifests itself as the extra dissipative work done by reorienting the solvent molecules’ dipoles as the charge moves through the medium. This long range phenomenon is also present within the bilayer, as *D*(*z*) for *tyr* ^+^ stays at a near constant value below that of *tyr* until well past the hydrophobic interface. Both *D*(*z*) values decrease at nearly the same rate while entering the headgroup region of the bilayer, but begin to diverge as they enter the hydrophobic acyl chain region. The unprotonated species shows an increase in diffusivity toward the center of the bilayer similar to that of O_2_ and water [28], while the *tyr* ^+^ value stays at ∼ 0.2 × 10^−5^ cm^2^/s. This is probably due to the hydration shell that accompanies *tyr* ^+^, keeping it in a more consistent environment throughout the hydrocarbon region. The increase in diffusion experienced by the unaccompanied *tyr* is likely due—as with O_2_ and water—to the greater availability of free volume toward the bilayer center [68].

The permeability calculation for tyramine is more complicated than for O_2_, as both species of the molecule need to be accounted for under the experimental conditions of interest. On their own, the two species show a huge difference in permeabilities (calculated using the curves from Figs 6 and 7 in Eq 1) spanning approximately ten orders of magnitude: *P*_*tyr*_ = (2 ± 1) × 10^−3^ cm/s and *P*_*tyr*^+^_ = (9 ± 11) × 10^−14^ cm/s, the latter being on the same order as Na^+^ permeating a lipid bilayer [69]. To account for the possible protonation states of tyramine, three approximations have been used that include both *tyr* and *tyr* ^+^ species in the calculation of the total permeability. The first approximation represents the limit for very slow interconversion between the two species along the entire reaction coordinate, and thus depends entirely on the bulk concentrations, i.e. *P*_*slow*_ = *f*_*tyr*_ *P*_*tyr*_ + *f*_*tyr*^+^_ *P*_*tyr*^+^_ where *f* is the fraction of the species in the bulk solvent. In our system at pH = 7.4, *f*_*tyr*_ = 0.008 and *f*_*tyr*^+^_ = 0.992. The second is for the opposite limit of very fast interconversion, where the resistance to permeability of the two species is analogous to electrical resistors in parallel. The third approximation (the *minimum resistance approximation* or MRA) is that used by Kessel *et al*. [70] (also see [71]), where the species with the lower of the two local resistance curves, *R*(*z*), is the one for which the permeability is calculated for that section of the reaction coordinate; this assumes that while in either state there is no interconversion. Physically, this approximates the deprotonation of *tyr* ^+^ to *tyr* at some point beyond the headgroup region. The *P* expressions for the latter two approximations are given in S2 Appendix, and in order to calculate their permeability the PMFs were offset in the bulk using the pK_*a*_ value for the amine. The PMF offset, denoted Δ*w*(0) in the supplementary information, varies from 2.27 kcal/mol to 6.09 kcal/mol for the pK_*a*_ range of 9–11.7. In the future it would be of interest to consider a more exact estimation of permeability P by taking into account the effect of the finite protonation and deprotonation rates. This will require modelling the reaction-diffusion behaviour of a tyramine molecule that is able to protonate and deprotonate while diffusing through the bilayer. One can model the proton transfer process assuming it is rate limited by proton diffusion. The protonation rate constant is then determined by proton diffusion, and the deprotonation rate constant can be determined from the protonation rate constant using detailed balance. In determining the latter rate constant from proton diffusion, the Grotthus—or cooperative proton diffusion—mechanism may be important, as seen from Fig 3B that shows water chains trailing the amine group of the *tyr* ^+^ molecule as it enters the bilayer from bulk solution.

**Fig 8:**
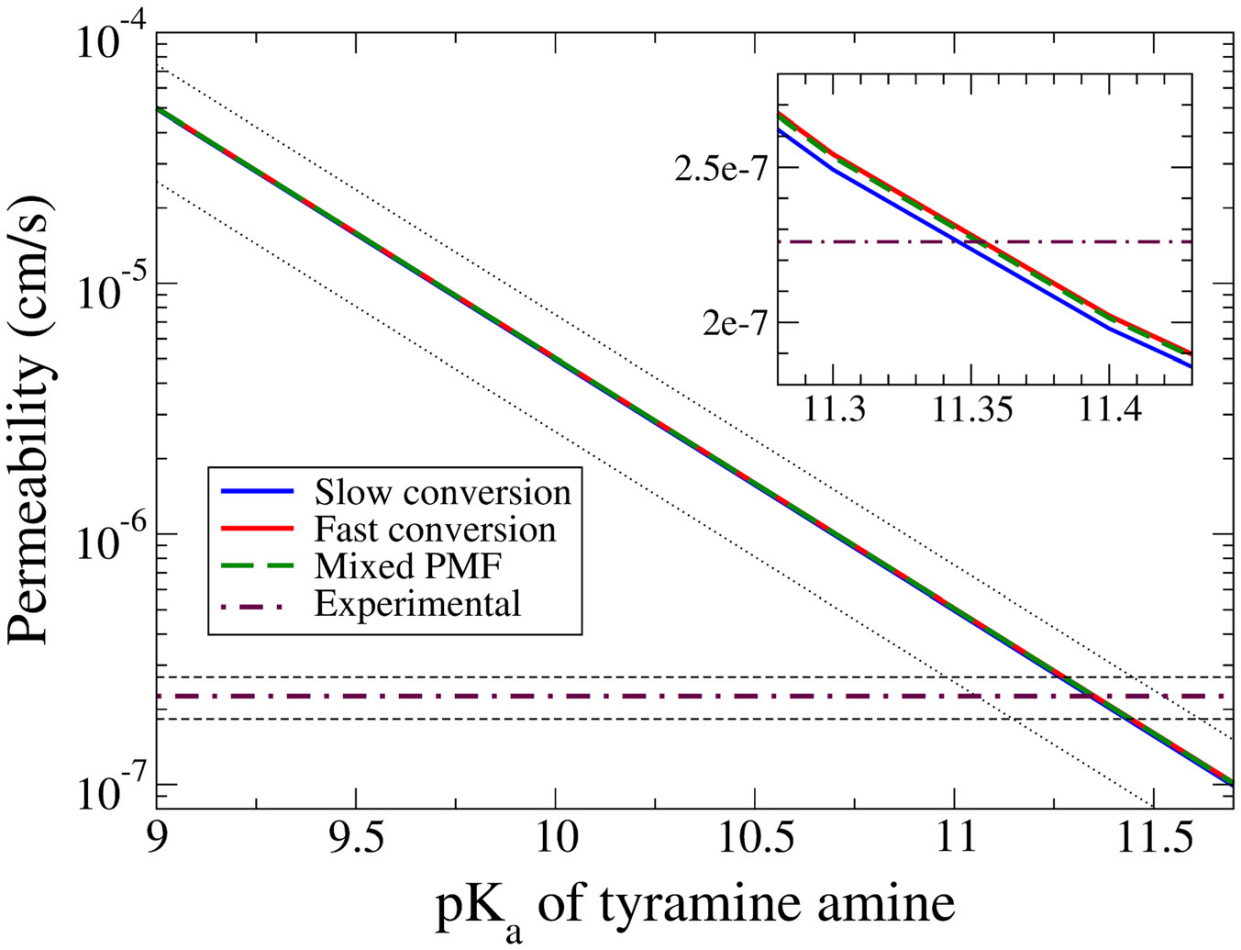
The permeability of tyramine calculated over the reported range of pK_*a*_ values for the amine group. The single standard error uncertainty for the simulated values (Slow conversion, Fast conversion, Mixed PMF) are plotted as black dotted lines, while the uncertainty for the experimental result is shown as black dashed lines. The inset is an expansion of the region where the plots converge.

Since all three approximations depend on the pK_*a*_ value of the amine group, and the reported experimental pK_*a*_ values vary between 9.3 and 10.9 [43–45, 72–74], the permeability calculations are shown in Fig 8 as a function of the amine pK_*a*_. All three approximations give nearly identical results, and can only be differentiated in the inset that shows the convergence region greatly magnified. The mathematical explanation for the similar results lies in the exponential weighting of the lower PMF for all of the approximations. Physically, the rate determining step in all three approximations is the time for a neutral tyramine to cross over its barrier. The experimental and simulated results converge at a pK_*a*_ ≃ 11.35, while the bounds of the uncertainties converge as low as 10.95. This is consistent with the measured and assigned pK_*a*_ values from [43–45] (see Table 1 in the ‘Discussion’), and is even more so if one accepts the above phase separation argument that approximately doubles the experimental result for *P*. It is possible that a more detailed calculation of the simulated *P* that involves the rate constants for the amine protonation reaction—and thus providing the fraction of time the permeant spends in each protonation state—could lead to a more accurate result, although this too would depend on the chosen pK_*a*_.

**Table 1:** Summary of experimental pK_*a*_ results from the literature.

Source	Year	Temperature (K)	amine pK_*a*_	phenolic pK_*a*_
Lewis [43]	1954	293	10.78	9.53, 9.77
Kappe and Armstrong [44]	1965	298	10.52	9.74
Armstrong and Barlow^a^ [45]	1976	298	10.6	9.23
Mack and Bönisch^b^ [72]	1979	298	9.17–10.86	9.17–10.86

^a^ Values for the 10 mM results.

^b^ The authors provide pK_*a*1_ and pK_*a*2_, but do not assign these to specific groups.

## Discussion

### Convergence of results

**Fig 9:**
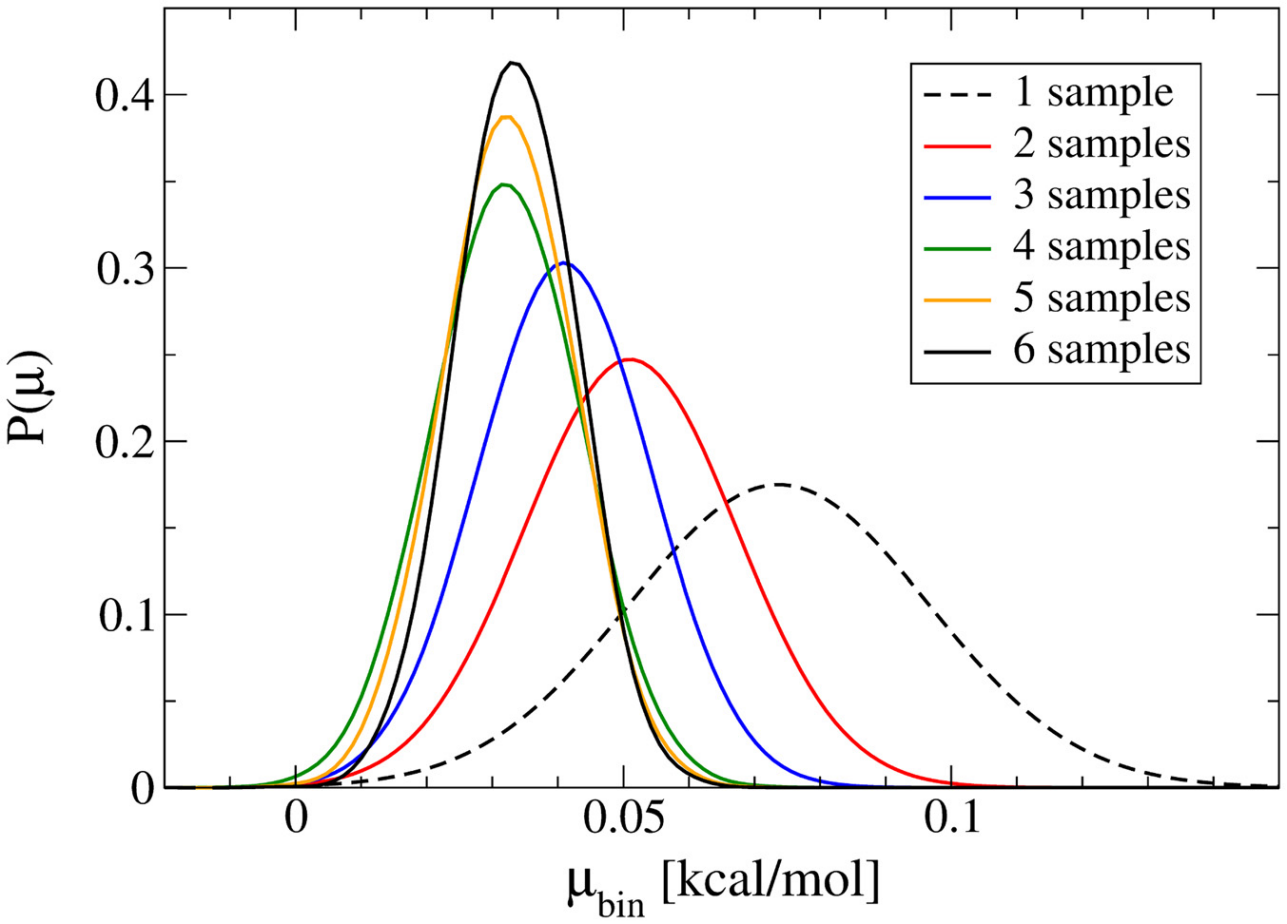
A representative example of the progression of probability densities through Bayesian inference, showing the bin at z = 2.0 Å for O_2_ at 323 K. All means started equally probable, and each plot represents one new sample of ⟨*W*_*rev*_⟩ (i.e. the evidence) added to the hypothesis. The predicted mean values of ⟨*W*_*rev*_⟩ shown in the distributions are denoted by *μ*_*bin*_.

**Fig 10:**
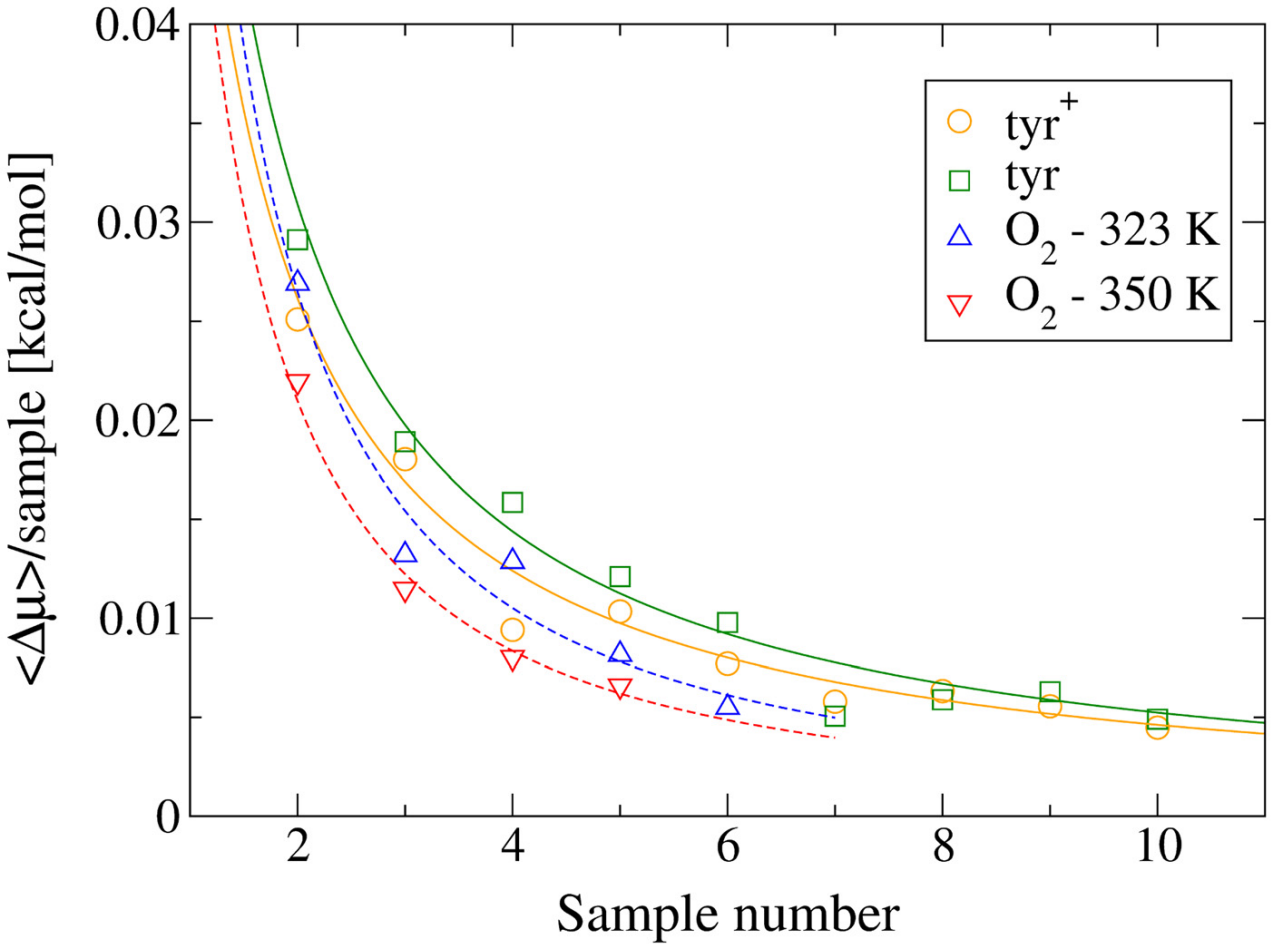
The average change in the mean, ⟨Δ*μ*_bin_⟩, as samples are added to the Bayesian hypothesis about the mean. For example, at *N* = 2, ⟨Δ*μ*_*bin*_⟩ = ⟨*μ*_*bin*_(2) − *μ*_*bin*_(1)⟩, and the most probable value from each Bayesian distribution was used for *μ*_*bin*_. The set averaged over was comprised of one bin every 1 Å (i.e. only every fifth bin was used). The fitted curve for *tyr* ^+^ was calculated without the outlier at Sample 4, while the curve for *tyr* was fitted without Sample 7.

Although Figs. C and D in S3 Appendix are instructional as to why multiple runs are necessary for these systems, the figures can also be misleading with regard to convergence. In order to quantify the convergence of our results, we performed a Bayesian analysis on the mean values of every fifth bin (i.e. every 1 Å). Fig 9 shows a representative example of how the means’ probabilities change (i.e. the hypothesis) with each new ⟨*W*_*rev*_⟩ value added (i.e. the evidence). The rate of convergence for O_2_ and tyramine is shown in Fig 10. In the figures, the mean value of ⟨*W*_*rev*_⟩ for a particular bin is represented by *μ*_*bin*_, Δ*μ*_*bin*_ is the change in the most probable value of *μ*_*bin*_ after more evidence has been added to the hypothesis, while the average of the latter quantity over many bins is denoted ⟨Δ*μ*_*bin*_⟩. The convergence is shown as a reduction in ⟨Δ*μ*_*bin*_⟩, and both sets of curves show that it is reduced to about 5 × 10^−3^ kcal/mol after all ⟨*W*_*rev*_⟩ have been added. This is a maximum of ∼ 7% of the standard error calculated for those bins, meaning the convergence of the means is well within the measured uncertainty.

It is interesting to note that the exponents for the fitted curves (of the form ⟨Δ*μ*_*bin*_⟩(*N*) ∝ *N* ^−*x*^) are 1.08 for tyramine and 1.33 for O_2_, meaning the curves converge faster than one would expect from independent random samples, i.e. ⟨Δ*μ*_*bin*_⟩(*N*) ∝ *N* ^−1^. This occurs because each value of ⟨*W*_*rev*_⟩ added as evidence is already an average over many samples. But since there were respectively 50 and 200 samples per bin, for increased convergence of the mean in a complex system it seems to be in general better to run multiple OFR runs in lieu of increasing the number of oscillations for an individual run (if increasing the number of oscillations also requires increasing the simulation time). At the same time, the uncertainty of each individual run still adds to the final uncertainty of the PMF, so one does not want to minimize the number of oscillations completely by using only one or two oscillations, but rather use a large enough value to give local uncertainties within a desired range. This also keeps the total OFR runs to a minimum, which could quickly become difficult to manage, especially when the equilibration time needed to create the separate runs is considered.

### Permeability

For the temperatures tested, the permeability of O_2_ through DPPC is at least as large as in bulk water (for the same thickness of TIP3P water, *P* ≈ 58 cm/s at 323 K, and *P* ≈ 77 cm/s at 350 K), allowing O_2_ to permeate the bilayer effectively without the need for any additional transport mechanisms. At 323 K the potential well within the lipid core of the bilayer acts as a permeation accelerant, more than doubling the bulk water value. At 350 K the well depth is insufficient to overcome the combination of the decreased rate of O_2_ diffusion within the bilayer and the increase in headgroup barrier width (compared to 323 K), leaving the overall permeability nearly the same as in the bulk solution. The idea of the bilayer acting as a permeation accelerant for hydrophobic molecules is an interesting phenomenon that is currently lacking an intuitive explanation. One might expect the potential well to act as a trap, as once the O_2_ is at the global minimum along *z* (within the context of the system), it becomes surrounded by potential barriers that must now be overcome to escape. Also counterintuitive, the ISD actually predicts a drop in permeability for the higher temperature system. This is likely due to the previously mentioned decrease in H-bonding in the surrounding water at higher temperatures, which could then lead to a decrease in the hydrophobic solubility of the non-polar permeant in the lipid core of the bilayer.

The work by Marrink and Berendsen [18] is the only theoretical work (known to the authors) to have calculated the permeability for O_2_, and they found *P* = 200 ± 500 cm/s at 350 K. This is consistent with our results, but is not useful for any comparison. Experimental values for the permeation of O_2_ were similarly sparse in the literature, but one work by Widomska *et al*. [75] did measure the permeability coefficients for O_2_ through a number of lipid systems. Two of the permeability coefficient values, *P* = 101 cm/s for the POPC/cholesterol mixture and *P* = 116 cm/s for the calf lens lipid mixture (both at *T* = 318 K), agree very well with this work. The POPC result from [75], *P* = 272 cm/s, is much higher than our 323 K result, but can be explained through the double bond in the oleoyl acyl chain. The kink in the chain that the double bond creates leads to looser packing than for fully saturated phospholipids, such as DPPC. The hypothesis is that with looser packing, there is more free volume and therefore greater diffusion of O_2_ through the bilayer. To further support this explanation, the permeability for the POPC/cholesterol mixture is much lower, presumably owing to the tighter packing (i.e. decrease in fluidity and reduction in free volume) afforded by the cholesterol. In the same work, Widomska *et al*. also showed that the greatest region of resistance to permeation occurs near the headgroups, agreeing with the PMFs from this work that both show a small barrier there (Fig 4).

For an accurate comparison of the tyramine experimental and simulation results from this work, the small temperature difference between these systems of 12 K (298 K and 310 K respectively) should be briefly discussed. In this temperature range, the shape of the PMF throughout the bilayer is highly dependent on—if not dominated by—the hydrophobic and hydrophilic effects. Since the dielectric constant of water is a monotonically decreasing function of temperature in this range [76], one would then expect both wells and barriers to decrease in size with increasing temperature, as in the case of O_2_. Thus, in contrast to O_2_, it is a reasonable expectation that the permeability of tyramine should *increase* with temperature. Together with the previously discussed phase separation of egg lecithin, the results displayed in Fig 8 probably converge at a lower pK_*a*_ than what is shown.

**Fig 11:**
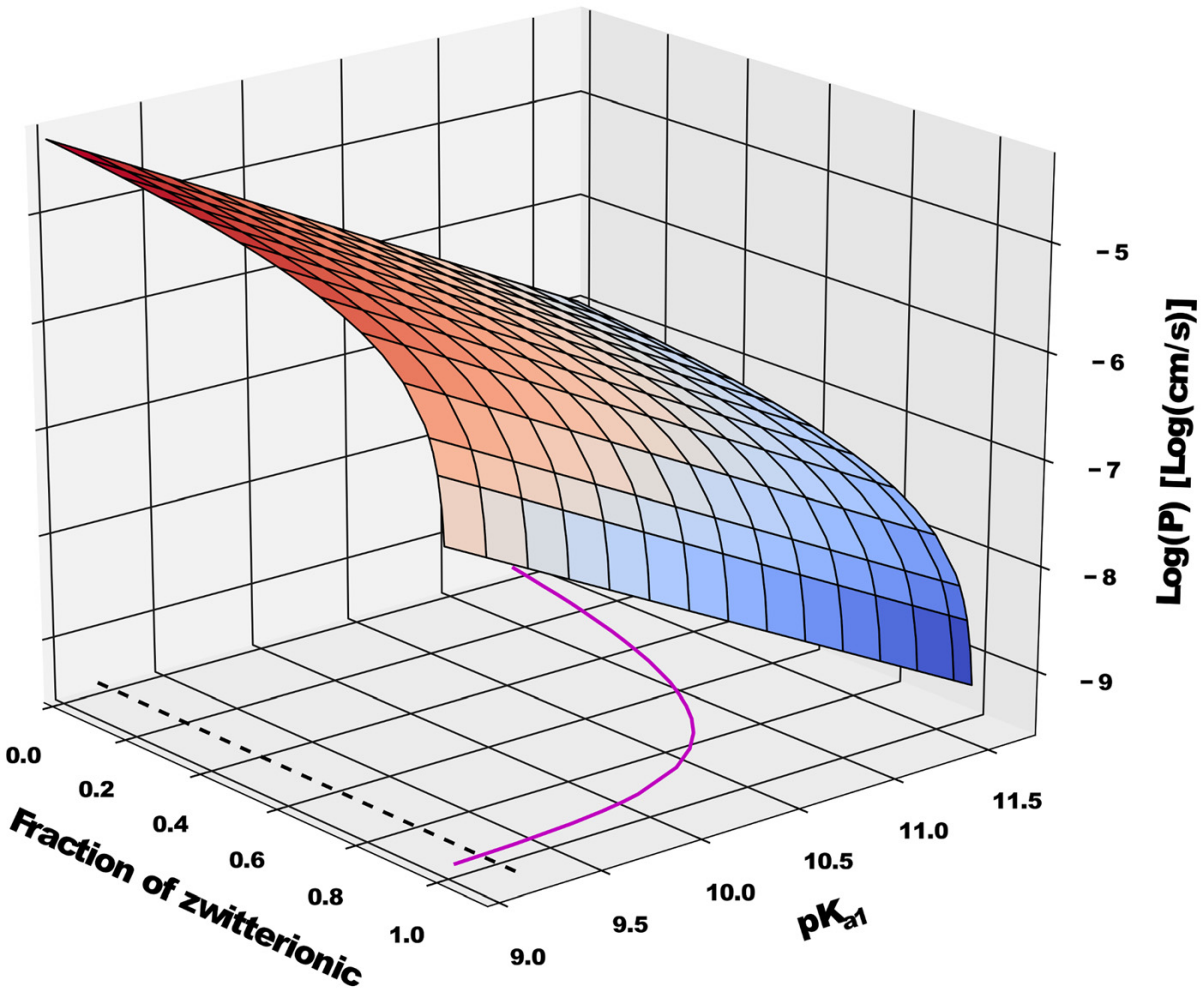
A surface plot of the simulation permeability results as a function of the macrodissociation constant pK_a1_, and the fraction of zwitterionic versus uncharged species, i.e. f_zw_ = 1 − f_un_. The solid purple line shows a contour of the surface for the experimental permeability value from this work, *P* = 2.26 × 10^−7^ cm/s, while the black dotted line shows the pK_*a*1_ value reported in Mack and Bönisch [72].

Other considerations aside, conversion of the experimental and simulated results suggest a high pK_*a*_ value for the amine group of tyramine. As previously mentioned, this is consistent with older measured pK_*a*_ values [43–45] (see Table 1), but more recent works suggest the values should be reversed. Shimamura *et al*. [73] report pK_*a*_ values of 9.3 (amine) and 10.4 (phenolic), while Partilla *et al*. [74] report a pK_*a*_ of 9.74 for the amine. However, it is unclear if these values were derived by the authors or if they were taken from an unreferenced source (it is possible that Partilla *et al*. mistakenly assumed the pK_*a*1_ from [44] represented the amine as it is closer to the pK_*a*_ of tyrosine’s amine group [77]. Here the terms pK_*a*1_ and pK_*a*2_ refer to the first and second pK_*a*_ values measured experimentally; more generally, the experimental pK_*a*_ values are often reported from lowest to highest, starting at pK_*a*1_ and going to pK_*ax*_, where *x* is index of the final pK_*a*_ measured for a particular molecule). To complicate matters, Mack and Bönisch [72] claim that the interplay bewteen the pK_*a*_ and both the amine and phenolic groups is more complex, and that simply assigning individual pK_*a*_ values is insufficient to describe the deprotonation events. They suggest that the pK_*a*1_ is in truth a *macrodissociation constant*, such that both groups actually begin to deprotonate at this pH—possibly to differing extents—while the pK_*a*2_ refers to the appearance of the negatively charged species due to both moieties being deprotonated. According to this picture, tyramine will exist as zwitterionic *and* uncharged species within the pH range of pK_*a*1_ and pK_*a*2_, with both species having their peak concentration at the midway point of the pK_*a*_ values, i.e. pH = (pK_*a*1_ + pK_*a*2_)/2. In order to determine how the current permeability results work under this hypothesis, Fig 8 has been extended to include a varying fraction of zwitterionic versus uncharged species, creating a permeability *surface* (Fig 11) with the amine pK_*a*_ replaced by pK_*a*1_. The contour in Fig 11 represents the experimental *P* from this work, and when the curve is at a pK_*a*1_ value of 9.17—the lower bound from Mack and Bönisch—the fraction of zwitterionic species is ∼ 95%. This strongly suggests that the phenolic group is the first to deprotonate. Combining this with the amine pK_*a*_ value at which the permeabilities converge (∼ 11), it still seems reasonable to assign the pK_*a*1_ to the phenolic group and the pK_*a*2_ to the amine, as the majority of authors have done in the past.

Another interesting result that stems from the detail provided by simulations, is the degree to which both the PMF and *D*(*z*) contribute to the permeability. It has long been argued [78–80] that the two limiting properties of a molecule’s permeability across a phospholipid bilayer are electrostatic charge and size. Our results agree with this hypothesis, and more specifically suggest that the polarity is largely responsible for the PMF curve—in agreement with the Born model—while the local diffusion coefficient correlates more strongly with the size of the molecule. The latter is not surprising as in a bulk fluid *D* ∝ *v* ^−*n*^, where *v* is the molecular volume and *n* ≈ 2/3 [81, 82], and numerous experimental [78, 80, 83–85] and *in silico* [86] works have shown an even more pronounced decrease in *D*(*v*) when crossing hydrophobic barriers. This explains why small monatomic ions have permeabilities on the order of 10^−11^–10^−14^ cm/s while a polar *tyr* has a higher permeability than a much smaller water molecule, as according to Eq 1 the PMF contributes exponentially to the permeability and *D*(*z*) only does so linearly. The only instance where the polarity also affects the diffusion is for highly polar molecules or molecules with a net charge, but this is most likely a side effect of the solvation layer effectively increasing the molecular volume.

Finally, since the partial charges used for tyramine were taken directly from DFT calculations, further parameterization of the *tyr* and *tyr* ^+^ molecules has the potential to improve the results. One should be careful, however, as parameterizing to agree with a metric such as the solvation free-energy could lead to inaccuracies within different media such as the bilayer. One way to circumvent this problem is to use a polarizable force-field such as AMOEBA [87], as an accurate parameterization for *tyr* and *tyr* ^+^ in water should also lead to accurate interactions in the bilayer. With computer power continuously increasing alongside the highly accelerated throughput provided by graphical processing units, this may soon be a viable option for the moderately simple systems simulated in this work.

## Concluding remarks

The OFR method—a type of nonequilibrium work method—has been used to obtain both the PMF, *w*(*z*), and the position dependent diffusion coefficient, *D*(*z*), for O_2_ through a DPPC bilayer, and uncharged and positively charged tyramine through a POPC bilayer. Model POPC bilayers are found to present a simple barrier for both forms of tyramine, with a barrier height of 21.0 kcal/mol for *tyr* ^+^ and 6.7 kcal/mol for *tyr*.

Oxygen permeability was calculated and found to be higher at 323 K (123 ± 35 cm/s) than at 350 K (78 ± 19 cm/s). These results fall into the same range and are consistent with previously reported simulation and experimental work for similar systems. However, this work is the first to observe the trend towards decreasing permeability as temperature is increased. The position dependent diffusion coefficient is shown to exhibit very little dependence on temperature and so the trend must be dominated by the temperature dependence of the PMF. The PMF well is much deeper for 323 K, perhaps due to the reduction in hydrophobic effects as the temperature is increased, and has the counter-intuitive effect of increasing the permeability. While leaving the well may seem to provide a barrier to permeation, in fact the effect of the well on permeability may be understood as the result of an increased probability for the molecule to reside in the hydrophobic core (twelve times higher at 323 K than at 350 K). The increase in residency would give the O_2_ more opportunity to overcome the energetic barrier presented by the opposite side of the well, in contrast to a molecule such as *tyr* ^+^ that would be able to diffuse away from the bilayer if it failed to overcome the barrier presented to it—this could perhaps be the cause of the increased permeability.

There was almost no measurable difference between the *D*(*z*) curves for O_2_ at the two temperatures, which is not surprising as the microenvironments for O_2_ in both systems were very similar. *D*(*z*) was calculated for both species of tyramine and found to vary over the range 0.2–1.0 × 10^−5^ cm^2^/*s*. The diffusion coefficient was found to depend more strongly upon location within the membrane than on the protonation state of the tyramine.

These data have been used to estimate the permeability coefficient for tyramine at 310 K as a function of the pK_*a*_ of the amine moeity, using the ISD equation [21]. Our simulated results were able to reproduce the experimental value, *P* = (2.26 ± 0.43) × 10^−7^ cm/s by constructing a model where the permeability due to protonated and uncharged forms was considered. The exponentiation of *w*(*z*) in Eq 1 means that the uncharged form, although far less available at physiologic pH, dominates the contributions to passive transport through the membrane. These results taken together lend strong support to the idea that, when carefully applied, MD simulation can be a powerful and accurate *quantitative* tool for predicting permeability and other properties of pharmacologically relevant molecules in model systems. By employing more realistic membrane models and with more complete experimental data, future work can hope to contribute significantly to the intelligent design of therapeutic agents.

## Supporting Information

S1 Appendix Atomic force parameters for the uncharged species of *p*-tyramine (CH_2_/OH). Contains: **Fig. A: A simple 2-D representation of the uncharged tyramine with the atoms labelled by their particle name.**
(PDF)

S2 Appendix Approximations for calculating the permeability using the ISD with multiple permeants. Contains: **Fig. B: The PMF of the positively and uncharged species of *p*-tyramine.** The dashed red line shows the PMF for the uncharged species shifted by the bulk deprotonation free-energy Δ*w*(0) = 2.3*k*_*B*_ *T*(pK_*a*_ − pH) = 5.6 kcal/mol, corresponding to a solution pH = 7.4 and a pK_*a*_ = 11.35 for the amine group of *p*-tyramine.
(PDF)

S3 Appendix Multiple run averaging of the OFR method. Contains: **Fig. C: The PMF of O_2_ across a model DPPC bilayer at 323 K (A) and 350 K (B), together with the reversible work, ⟨W_rev_⟩, from all individual OFR runs.** All curves are zeroed in bulk water, at *z* = 30.4 Å from the bilayer center (*z* = 0). The PMFs are calculated from the individual runs using the BD-FDT for each bin separately. **Fig. D: The PMF of *tyr* and *tyr*^+^ across a model POPC bilayer, together with the reversible work, ⟨W_rev_⟩, from all ten individual OFR runs.** All curves are zeroed in bulk solution, at *z* = 38.4 Å from the bilayer center (*z* = 0). The PMF is calculated from the individual runs using the BD-FDT for each bin separately.
(PDF)
