# Supplementary material for: A Permeability Study of O2 and the Trace Amine p-Tyramine through Model Phosphatidylcholine Bilayers
Source: PLoS One. 2015 Jun 18;10(6):e0122468. doi: 10.1371/journal.pone.0122468 (PMC4472697; doi:10.1371/journal.pone.0122468)
Supplement: S1 Appendix — Contains: Fig. A: A simple 2-D representation of the uncharged tyramine with the atoms labelled by their particle name. (PDF) [file pone.0122468.s001.pdf]

S1 Appendix. Atomic force parameters for the uncharged species of *p*-tyramine (CH_2/OH).

Table A. All of the coefficients used to calculate forces for the uncharged species of tyramine, including the partial charges and the two Lennard-Jones (LJ) parameters for the well depth, ϵ, and radius, $r_{min}/2$, (the LJ parameters are taken from the standard CHARMM27 parameter file)

Particle name	Element	CHARMM type	Charge (unitary)	LJ - ϵ (kcal/mol)	LJ - $r_{min}/2$ (Å)
N	N	NH2	-1.0279	0.2000	1.8500
HN1	H	HC	0.3708	0.0460	0.2245
HN2	H	HC	0.3708	0.0460	0.2245
CA	C	CT2	0.3379	0.0550	2.1750
HA1	H	HA	-0.0132	0.0220	1.3200
HA2	H	HA	-0.0132	0.0220	1.3200
CB	C	CT2	-0.0547	0.0550	2.1750
HB1	H	HA	0.0527	0.0220	1.3200
HB2	H	HA	0.0527	0.0220	1.3200
CG	C	CA	-0.0155	0.0700	1.9924
CD1	C	CA	-0.2007	0.0700	1.9924
HD1	H	HP	0.1610	0.0300	1.3582
CE1	C	CA	-0.2196	0.0700	1.9924
HE1	H	HP	0.1545	0.0300	1.3582
CZ	C	CA	0.3072	0.0700	1.9924
OH	O	OH1	-0.5423	0.1521	1.7700
HH	H	H	0.3843	0.0460	0.2245
CD2	C	CA	-0.2007	0.0700	1.9924
HD2	H	HP	0.1610	0.0300	1.3582
CE2	C	CA	-0.2196	0.0700	1.9924
HE2	H	HP	0.1545	0.0300	1.3582

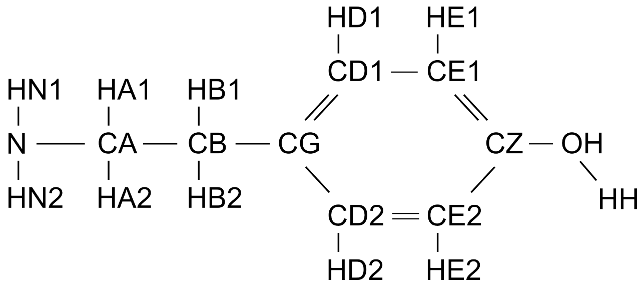

Figure A. A simple 2-D representation of the uncharged tyramine with the atoms labelled by their particle name.
