# Supplementary material for: A Permeability Study of O2 and the Trace Amine p-Tyramine through Model Phosphatidylcholine Bilayers
Source: PLoS One. 2015 Jun 18;10(6):e0122468. doi: 10.1371/journal.pone.0122468 (PMC4472697; doi:10.1371/journal.pone.0122468)
Supplement: S2 Appendix — Contains: Fig. B: The PMF of the positively and uncharged species of p-tyramine. The dashed red line shows the PMF for the uncharged species shifted by the bulk deprotonation free-energy Δw(0) = 2.3k B T(pKa − pH) = 5.6 kcal/mol, corresponding to a solution pH = 7.4 and a pKa = 11.35 for the amine group of p-tyramine. (PDF) [file pone.0122468.s002.pdf]

S2 Appendix. Approximations for calculating the permeability using the ISD with multiple permeants.

As shown in the Introduction, the *inhomogeneous solubility-diffusion* (ISD) model can be use to calculate the permeability by using the *potential of mean force* (PMF), $w(z)$, and the position dependent diffusion coefficient, $D(z)$, as follows:

$$R = \frac{1}{P} = \int_0^L dz \frac{e^{\beta w(z)}}{D(z)} \ , \tag{S2.1}$$

where the result R is the *resistance to permeability.* The ISD is only capable of calculating the permeability for a single molecule or particle, so when multiple species contribute to the total permeability (as for p-tyramine), there need be a way to evaluate the contribution from each of these species. This contribution is dependent on their calculated P values from the ISD, their relative concentrations in solution prior to permeation (which is usually a direct result of the solution pH), and the reaction rate constants between species as a function of the depth of the medium being permeated, $k_f(z)$ and $k_r(z)$, for the forward and reverse reactions respectively. The latter refers to a complex interaction that is difficult to simulate using molecular dynamics where no chemical bonds are ever broken, and so approximations to the total P value have either ignored the reaction rate constants or used their extrema in lieu of the actual functions.

Two approximations that use the limiting behaviour of the reaction rates are what we have called the *slow interconversion* and the *fast interconversion.* The slow interconversion represents the case where the reaction rates are so slow that all species of the molecule will always permeate the medium prior to any conversion to another species. For the p-tyramine species studied in this work, this means that the positively charge species will never deprotonate, and the uncharged species will never gain a proton. Thus the net permeability of all the species is simply a sum of the individual permeabilities weighted by their fractional probability of existing in the bulk medium,

$$P = \sum_{i=1}^{n} f_i P_i \ . \tag{S2.2}$$

The fast interconversion limit implies the opposite extreme for the reaction rates, being that the rates are so high the species will interconvert immediately upon impetus to do so. This means that even for nonequilibrium trajectories, each species will exist throughout the bilayer in proportion to the Boltzmann weight of their respective PMF. In order for this limit to be possible, there is a strong assumption that

it is always immediately possible for a chemical change to occur, meaning the raw materials for the change are ever present. In the case of p-tyramine, this means there would always be a proton donor available for the uncharged species, and a proton acceptor for the positively charged species. Through a treatment of the ISD with the Fokker-Plank equation (to be presented in a future article, as the theoretical scaffolding required is outside the scope of the current work), the following result can be obtained for this interconversion limit that is analogous to resistance in circuits:

$$R = \int_0^L dz \left[\sum_{i=1}^{n} e^{-\beta w_i(z)} D_i(z) \right]^{-1} = \int_0^L dz \left[\sum_{i=1}^{n} \frac{1}{R_i(z)} \right]^{-1} . \tag{S2.3}$$

The final approximation has two slightly different forms – though both of which assume that P is given by only a single contributor at any point along the reaction coordinate, z, while the remaining species are ignored. The first assumes the greatest contributor to the permeability is always the most abundant species, i.e. the species with the lowest PMF

$$P = \left[\int_0^L \frac{\min(e^{\beta w_i(z)})}{D_i(z)} dz \right]^{-1} . \tag{S2.4}$$

This form is attractive from a chemical perspective as the position along z where the PMFs cross is also the most likely location for the chemical change between species to occur. This is not, however, the form that correctly assumes the greatest contribution to the permeability, as it completely ignores the diffusion. Therefore, the second form assumes that the greatest contribution is from the species with the lowest *resistivity*, $R(z)$,

$$P = \left[\int_0^L \min(R_i(z)) dz \right]^{-1} . \tag{S2.5}$$

To implement Eqs. S2.3 to S2.5, the bulk concentration of each species is taken into account by shifting the PMFs according to their pK_a values. Fig. B shows the PMF of the uncharged species shifted by $\Delta w(0)$ to account for the species concentration at a specific pH and pK_a value for the amine group. The slow interconversion simply uses the original PMFs that are zeroed in the bulk water, shown in Fig. B by the solid blue and red lines.

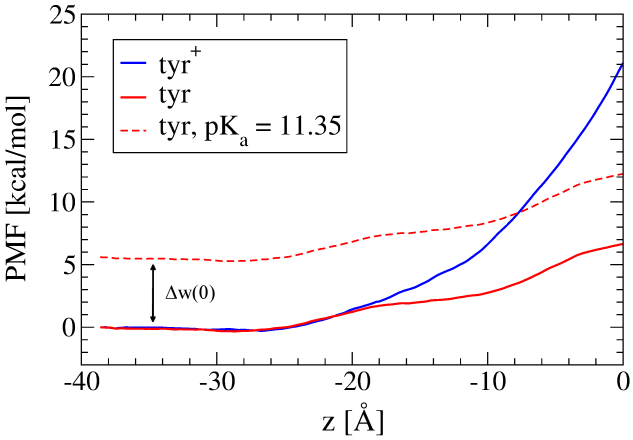

Figure B. The PMF of the positively and uncharged species of *p*-tyramine. The dashed red line shows the PMF for the uncharged species shifted by the bulk deprotonation free-energy $\Delta w(0) = 2.3 k_B T(\mathrm{pK}_a - \mathrm{pH}) = 5.6\,\mathrm{kcal/mol}$, corresponding to a solution pH = 7.4 and a $\mathrm{pK}_a = 11.35$ for the amine group of *p*-tyramine.
