# Supplementary material for: A Permeability Study of O2 and the Trace Amine p-Tyramine through Model Phosphatidylcholine Bilayers
Source: PLoS One. 2015 Jun 18;10(6):e0122468. doi: 10.1371/journal.pone.0122468 (PMC4472697; doi:10.1371/journal.pone.0122468)
Supplement: S3 Appendix — Contains: Fig. C: The PMF of O2 across a model DPPC bilayer at 323 K (A) and 350 K (B), together with the reversible work, ⟨Wrev⟩, from all individual OFR runs. All curves are zeroed in bulk water, at z = 30.4 Å from the bilayer center (z = 0). The PMFs are calculated from the individual runs using the BD-FDT for each bin separately. Fig. D: The PMF of tyr and tyr+ across a model POPC bilayer, together with the reversible work, ⟨Wrev⟩, from all ten individual OFR runs. All curves are zeroed in bulk solution, at z = 38.4 Å from the bilayer center (z = 0). The PMF is calculated from the individual runs using the BD-FDT for each bin separately. (PDF) [file pone.0122468.s003.pdf]

S3 Appendix. Multiple run averaging of the OFR method.

Figs. C and D include results from the individual runs, shown as cumulative reversible work curves, $\langle W_{rev} \rangle$. The $\langle W_{rev} \rangle$ curves clearly show that a single run is not sufficient to sample the relevant phase space for the reaction coordinate, and could lead to mis-evaluations as high as 2.5 kcal/mol at the bilayer center for O_2, and ~ 5 kcal/mol for *tyr*. Conversely, from all the $\langle W_{rev} \rangle$ curves it is clear that a wide range of the relevant phase space has been sampled. The disparity between curves is large enough, however, that one might believe there were not enough runs performed to achieve convergence for the PMFs. But what seems to be undersampling is merely an artifact of the comparison method used to show the spread among individual curves. This is because each final PMF curve is not calculated using the cumulative values of $\langle W_{rev} \rangle$ shown, but rather by using the $\langle W_{rev} \rangle$ from the individual bins. See the 'Discussion' section of the text for a Bayesian analysis of the PMF convergence.

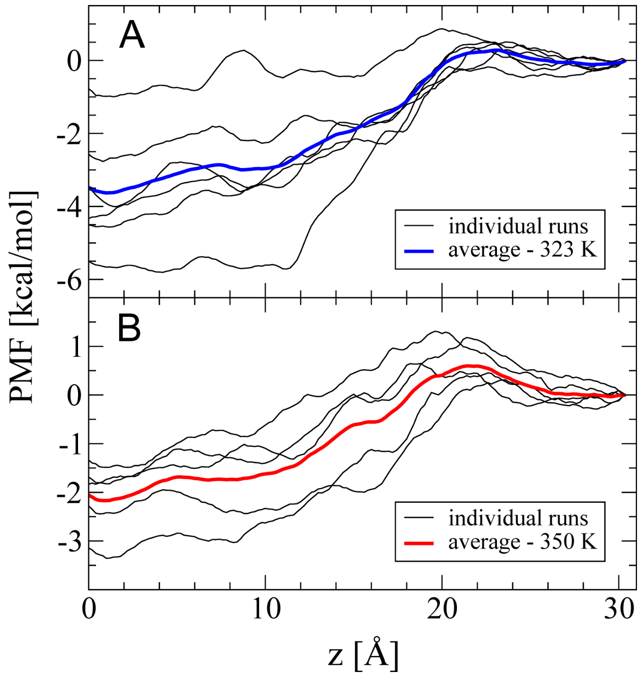

Figure C. The PMF of O_2 across a model DPPC bilayer at 323 K (A) and 350 K (B), together with the reversible work, $\langle W_{rev} \rangle$, from all individual OFR runs. All curves are zeroed in bulk water, at $z = 30.4$ Å from the bilayer center ($z = 0$). The PMFs are calculated from the individual runs using the BD-FDT for each bin separately.

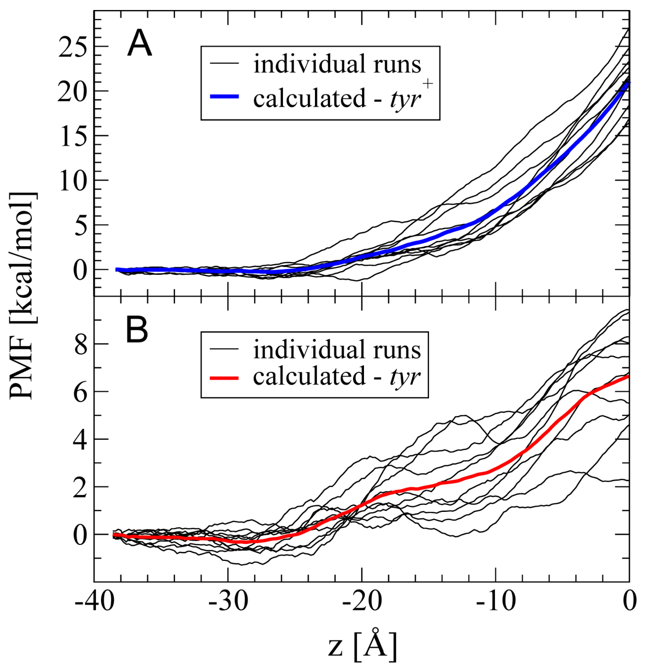

Figure D. The PMF of *tyr* and *tyr*$^+$ across a model POPC bilayer, together with the reversible work, $\langle \mathbf{W_{rev}} \rangle$, from all ten individual OFR runs. All curves are zeroed in bulk solution, at $z = 38.4$ Å from the bilayer center ($z = 0$). The PMF is calculated from the individual runs using the BD-FDT for each bin separately.
